# Quantifying Cell Fate Decisions for Differentiation and Reprogramming of a Human Stem Cell Network: Landscape and Biological Paths

**DOI:** 10.1371/journal.pcbi.1003165

**Published:** 2013-08-01

**Authors:** Chunhe Li, Jin Wang

**Affiliations:** 1 Department of Chemistry and Physics, State University of New York at Stony Brook, Stony Brook, New York, United States of America; 2 State Key Laboratory of Electroanalytical Chemistry, Changchun Institute of Applied Chemistry, Chinese Academy of Sciences, Changchun, Jilin, China; University of Minnesota, United States of America

## Abstract

Cellular reprogramming has been recently intensively studied experimentally. We developed a global potential landscape and kinetic path framework to explore a human stem cell developmental network composed of 52 genes. We uncovered the underlying landscape for the stem cell network with two basins of attractions representing stem and differentiated cell states, quantified and exhibited the high dimensional biological paths for the differentiation and reprogramming process, connecting the stem cell state and differentiated cell state. Both the landscape and non-equilibrium curl flux determine the dynamics of cell differentiation jointly. Flux leads the kinetic paths to be deviated from the steepest descent gradient path, and the corresponding differentiation and reprogramming paths are irreversible. Quantification of paths allows us to find out how the differentiation and reprogramming occur and which important states they go through. We show the developmental process proceeds as moving from the stem cell basin of attraction to the differentiation basin of attraction. The landscape topography characterized by the barrier heights and transition rates quantitatively determine the global stability and kinetic speed of cell fate decision process for development. Through the global sensitivity analysis, we provided some specific predictions for the effects of key genes and regulation connections on the cellular differentiation or reprogramming process. Key links from sensitivity analysis and biological paths can be used to guide the differentiation designs or reprogramming tactics.

## Author Summary

Cellular differentiation and reprogramming have been extensively studied using experimental methods. We developed a landscape and kinetic path approach to explore the global stability of a stem cell developmental network. The cell fates are quantified by the basins of attractions of the underlying landscape. The developmental process can be quantitatively described and uncovered by the biological paths on the landscape from the progenitor state to the differentiation state. This allows us to trace the underlying detailed kinetic process and obtain the recipe for engineering differentiation and reprogramming. By quantifying the landscape topography by the barrier heights and dynamic transition speed, we can evaluate the stability and kinetics of cell fate decision making process of the development and reprogramming. The global sensitivity analysis provided predictions about the effects of the key genes and regulation links of the network on the stability of differentiation and reprogramming process. This can be tested in the experiments. Results from sensitivity analysis and biological paths acquired can be used to guide the differentiation designs or reprogramming tactics.

## Introduction

Human pluripotent stem cells have the potential to produce any tissues in the body, which provides the motivation for many researchers to investigate the cellular reprogramming. Recently some research on cellular reprogramming show that the transformation from somatic cells to induced pluripotent stem cells (iPSC) or between different differentiation cell types can be implemented by manipulating a few key genes [1]–[6]. These results provide hints for the stem cell models to be applied to the regenerative medicine.

However, it is still challenging to generate and manipulate human pluripotent stem cells before practical applications to human healths. The efficiency of current cellular reprogramming techniques is often low and the molecular mechanism of cellular differentiation and reprogramming is still not very clear so far. This might be one of the main hurdles for iPSC to be applied for therapy. Therefore, understanding mechanisms of cellular differentiation and reprogramming as well as finding the optimal reprogramming pathway become very important for the application of iPSC. This requires a systematic and global approach to explore underlying gene regulatory networks with marker genes and mutual regulations between them.

The epigenetic landscape concept has been proposed to explain the development and differentiation of the cells as a metaphor [7], and provided a quantitative way of understanding the dynamics of gene regulatory system that drive cell development. This picture has been quantitatively realized through exploration of the global nature of the network in terms of probabilistic landscape framework [8]–[17]. The state space of gene regulatory networks contains states with different gene expression patterns (such as embryonic stem cell marker gene NANOG and OCT4) in the cell, which further determines different cellular phenotypes. Using landscape framework, cell types are represented by basins of attractions on the landscape, which reflect the probability of appearance of different cell types. States with lower potential or higher probability represent attractor states or biological functional states, surrounded by the basin of attraction. So, the biological process such as cellular differentiation or lineage commitment can be understood as the transition from an attractor state to another one in the gene regulatory network state space. By quantifying the topography of the potential landscape in terms of barrier heights and transition rates, we can explore the global stability, kinetic paths and kinetic speeds of cell fate decision making process.

We will explore the underlying landscape of a human stem cell developmental and differentiation network with 52 gene nodes by constructing the corresponding chemical reaction rate equations to explore its global properties, uncover the functional mechanism of transition between stem cell states and differentiation states. The barrier heights separating basins of attractions and the transition rates serve as the quantitative measure for global stability and kinetics of cell fate decision making process from one cell fate attractor to another representing different cell types. We further quantify the kinetic paths for the differentiation and reverse differentiation process (reprogramming). We show that both potential landscape and probabilistic flux determine the dynamics of the developmental system. The force from the curl flux leads the kinetic paths of the system deviating from the steepest descent gradient one of potential. As a consequence, the differentiation path and the reprogramming path are irreversible. By identifying the differentiation and reprogramming paths, we can quantitatively trace the important states along the paths. Based on this, we can find out the detailed kinetic process realizing the differentiation and reprogramming. By the global sensitivity analysis of parameters or connections between genes, we will quantitatively predict which connection links or nodes (genes) are critical to cellular differentiation or reprogramming, which can be directly tested from the experiments. Through the analysis on the underlying landscape, we can also understand more clearly the mechanism of differentiation and reprogramming as well as the sensitivity of the parameters or links on the stability of the stem cell system. The biological paths we acquired can be used to guide the design of new differentiation or reprogramming tactics. This also provides a way to explore the biological paths for high dimensional systems or large networks.

## Results/Discussion

### Landscape, Flux, and Kinetic Path for Development Network

**Figure 1:**
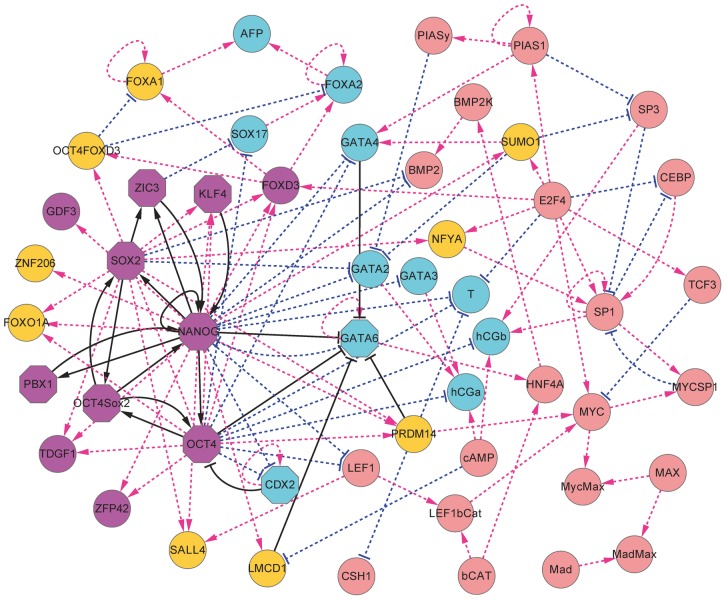
The diagram for the stem cell developmental network including 52 gene nodes and their interactions (arrows represent activation and perpendicular bars represent repression). The magenta node represent 11 marker genes for the pluripotent stem cell state, cyan nodes represent 11 marker genes for the differentiation state, and the yellow nodes represent genes activated by the stem cell marker genes. The solid black links represent the key links found by the global sensitivity analysis, and the octagon shape nodes represent key stem cell and differentiation markers found by global sensitivity analysis.

We obtained the steady state probability distribution and potential landscape of the 52 gene stem cell regulatory network system (Figure 1) [18] by self consistent mean field approximation, according to 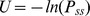 [8], [10]–[15]. Here, 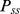 represents the probability distribution of the steady state, and 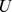 is the dimensionless potential energy. Therefore, 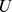 directly reflects the steady state probability. Figure S1 gives a flowchart for the methods that we employed in this work.

The time evolution the dynamical systems are governed by the diffusion equations. Given the system state 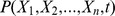, where 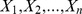 is the concentration or populations of molecules or species, we expected to have N-coupled differential equations, which are difficult to solve. Following a self consistent mean field approach [8], [12], [19], we split the probability into the products of individual ones: 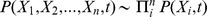 and solve the probability self-consistently. This effectively reduces the dimensionality from 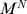 to 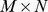, and thus makes the problem computationally tractable. For the multi-dimensional system, it is still hard to solve diffusion equations directly. We start from moment equations and assume specific probability distribution based on physical argument, which means that we give some specific connections between moments. In principle, once we know all moments, we can construct the probability distribution. Here we use gaussian distribution as approximation (two moments are needed, mean and variance). Therefore, the evolution of probabilistic distribution for each variable can be acquired after solving the moment equations (the mean and variance) based on gaussian approximation approach (See Methods for detailed self consistent approximation method for obtaining landscape). In this work, we acquired 52 dimensional probability distribution. For a 52-dimensional system, it's hard to visualize the landscape. So we integrated out the other 50 variables and left two key variables NANOG and GATA6, then projected the landscape to a 2-dimensional state space (NANOG and GATA6). The reason that we chose the variable NANOG and GATA6 is because that NANOG is a major stem cell marker gene, GATA6 is a major differentiation marker gene, and the regulation dynamics of the 52 nodes network is mainly determined by the mutual repression of NANOG and GATA6 and the mutual repression between OCT4 and CDX2. Choosing other major genes for presentation (such as any 2 genes from NANOG, GATA6, OCT4 and CDX2) will give the similar bistable landscape picture.

**Figure 2:**
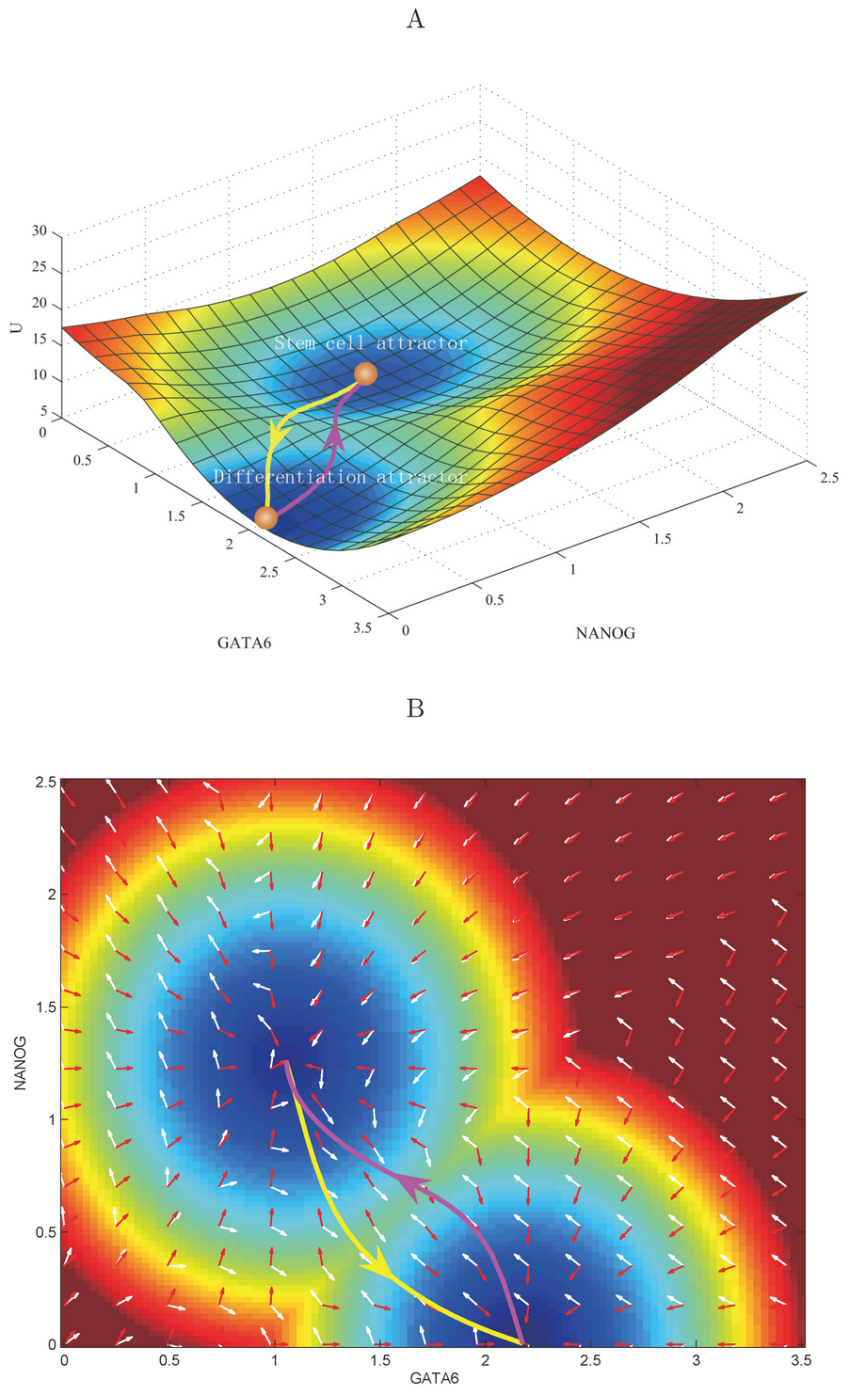
A bistable landscape picture for the stem cell network. Parameters are specified as: 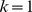 (degradation), 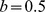 (repression), 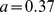 (activation), and diffusion coefficient 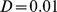. (A) Three dimensional landscape and dominant kinetic paths. The yellow line represents developmental path, and the magenta line represents reprogramming path. (B) Two dimensional dominant kinetic path and flux on the landscape. The white arrows represent the direction of flux, and the red arrow represent the direction of the negative gradient of potential energy.

Figure 2 shows two-dimensional and three-dimensional landscape in transcription factor expression level NANOG/GATA6 state space and the kinetic paths for the system. In Figure 2(A), we can see clearly that there are two stable states or basins of attractions on the landscape (bistability). One of them represents the pluripotent stem cell state, which has higher expression of stem cell marker—such as OCT4, NANOG and lower expression of differentiation marker genes—such as GATA6; and the other stable state represents the differentiation state, corresponding to lower expression of stem cell marker genes, and higher expression of differentiation marker genes. Based on our path integral method [13], [20], we also acquired the quantitative developmental path from the stem cell state to the differentiation state (and the reprogramming path from the differentiation state to the stem cell state). Here, parameters are chosen in order to obtain relatively balanced two states (stem cell state and differentiation state). Specifically, we set degradation constant 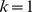, activation constant 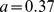, and repression constant 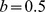 (See Methods Section).

In order to exhibit the landscape of the complete 52 dimensional network, we also used Langevin dynamics method to obtain landscape (Figure S2 in supporting information). For a 52 dimensional system, for visualization, we harnessed 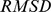 (root mean squared distance) as the coordinate to reduce the dimensionality to 2 dimension (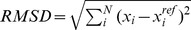, 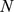 is the number of variables, and 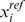 is the reference state, here we chose two potential minima as the reference states). 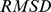 represents the distance between a state point and reference point in state space. In this way, from 52-dimensional trajectory, we can generate two new coordinates 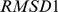 and 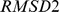, separately representing the distance from a state point to the reference state 1 (the potential minimum of stem cell attractor) and the reference state 2 (the potential minimum of differentiation state attractor). We can find that the landscapes using RMSD method based on Langevin dynamics (Figure S2 in supporting information) possess the similar dynamics compared with using NANOG and GATA6 as the coordinates (Figure 2) based on the self consistent approximation. This shows that the two dimensional projection of landscape in NANOG and GATA6 state space can reflect the major dynamics of the full 52-dimensional gene network.

We also showed the probabilistic flux of the stem cell system on the landscape (Figure 2(B)). The white and red arrows respectively represent the direction of probabilistic flux and the negative gradient of the potential energy. The dynamics of the developmental system is determined by both the force from the gradient of potential and the force from the curl flux [11]. The force from the curl flux leads the paths of the system deviating from the steepest descent path calculated from gradient of potential, therefore, as we can see the two kinetic paths of differentiation and reprogramming are irreversible (yellow line and red line are not identical), which was indicated in both adiabatic and non-adiabatic mechanisms for the stem cell developmental motifs with only two genes [13], [14]. Here, we can see the basic picture holds true for the gene network at the realistic level with 52 genes.

**Figure 3:**
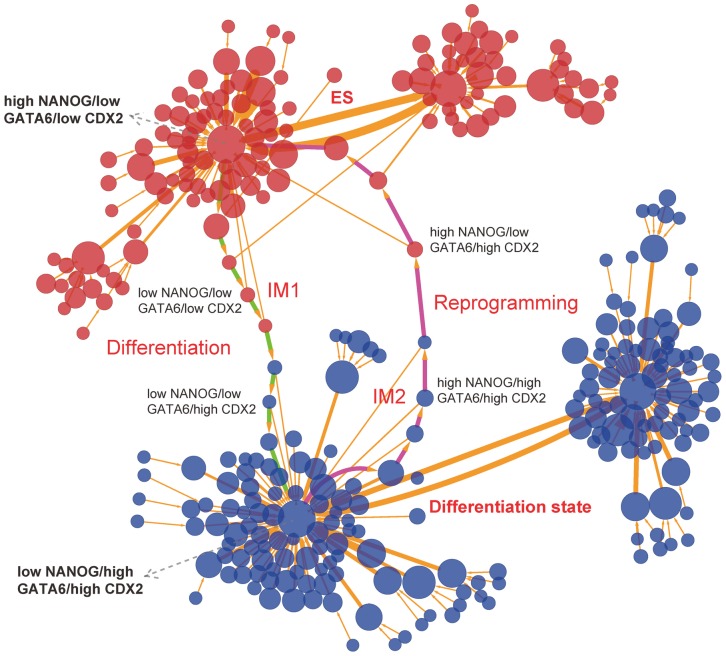
Differentiation and reprogramming process represented by 313 nodes (every node denotes a cell state, characterized by expression patterns of the 22 marker genes) and 329 edges (paths). The sizes of nodes and edges are proportional to the occurrence probability of the corresponding states and paths, respectively. Red nodes represent states which are closer to stem cell states in terms of gene expression pattern, and blue nodes represent states which are closer to differentiation states. The green and magenta paths denote dominant kinetic paths from path integral separately for differentiation and reprogramming. Here, we set a probability cutoff to decrease the number of states and paths, i.e. we only demonstrate the states and paths with higher probability. The largest red node (high NANOG/low GATA6/low CDX2) represents most major ES state (stem cell state), and the largest blue node (low NANOG/high GATA6/high CDX2) represents most major differentiation state. IM1 represents a intermediate state (low NANOG/low GATA6/low CDX2), and IM2 represents another intermediate state (high NANOG/high GATA6/high CDX2).

The landscape in Figure 2 only is a 2-dimensional projection of the whole 52 dimensional state space. In order to demonstrate the cell states and the transitions between different cell types in the complete state space, we projected the expression level of the 22 marker genes to binary states (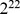 cell states). Here, to analyze the dynamics of the system, we chose the key 22 marker genes to explore the underlying landscape and transition jumps between two nodes based on Langevin dynamics. The first reason choosing the 22 maker genes is that the 52 dimensional state space is huge, and it will have 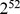 states even in the discrete form, which cannot be easily handled computationally. Another reason is that we believe using key 22 maker genes can capture major regulatory dynamics or paths without losing the essential information, since our purpose is to explore the dynamical mechanism of the stem cell differentiation system. For example, the stem cell state is represented by the binary number 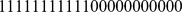 (representing expression level from gene 1 to gene 22, 1 for high expression, 0 for low expression), and for the differentiation state, it is represented by 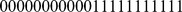. Figure 3 (see Methods Section for detailed methods) shows the differentiation and reprogramming process represented by 313 cell states (nodes, characterized by expression patterns of the 22 marker genes) and 329 transition jumps (edges) between the different cell states (produced by Cytoscape [21]). The sizes of nodes and edges are respectively proportional to the occurrence probability of the corresponding states and paths. Red nodes represent states which are closer to stem cell states, and blue nodes represent states which are closer to differentiation states. In particular, we displayed the 22 dimensional kinetic paths (biological paths) from path integral methods (see methods for the details of path integral), which are shown as green and magenta paths separately for differentiation and reprogramming process (see Table S4 and Table S5 for detailed paths). We can see that the paths for differentiation and reprogramming are irreversible. The irreversibility of the paths implies the time asymmetry which may point out the direction of the development.

From Table S4, monitoring the differentiation process according to certain vital marker genes NANOG (column 3), GATA6 (column 16) and CDX2 (column 22), we can see that the differentiation process experiences a transition from the stem cell state (high NANOG/low GATA6/low CDX2) to a intermediate state (IM1, low NANOG/low GATA6/low CDX2), and then to another intermediate state (low NANOG/low GATA6/high CDX2), and eventually to the differentiation state (low NANOG/high GATA6/high CDX2). This indicates the importance of NANOG to the maintenance of pluripotency. For differentiation proceeding, the cell needs to firstly impair the expression of NANOG, further downregulate other stem cell marker genes which are promoted by NANOG, and finally reach the differentiation state (GATA6 dominant). For the reprogramming path in Table S5, we can see that the cell experiences a transition from the differentiation state (low NANOG/high GATA6/high CDX2), to an intermediate state (IM2, high NANOG/high GATA6/high CDX2), to another intermediate state (high NANOG/low GATA6/high CDX2), and finally to the stem cell state (high NANOG/low GATA6/low CDX2). This might imply that in the reprogramming process the cell first opens the key stem cell marker genes NANOG by the change of regulation strength between key maker genes, then other stem cell marker genes gradually acquire high expression level due to the activation regulation of NANOG to them. Finally the cell reach the stem cell state, because the stem cell marker genes which have been activated repress strongly the differentiation marker genes (such as GATA6 and CDX2). The biological paths can be validated by related experiments [18], and we expect that it can be used to guide the design of new strategies for cellular differentiation and reprogramming.

### Barrier Height and Kinetics for Developmental Network

To quantify the global stability of the stem cell network in terms of landscape topography, we define global barrier height, representing potential difference between two attractor minimums and the saddle point on landscape. We define 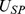 as the potential energy difference between the pluripotent stem cell state and the saddle point, 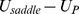, and 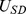 as the potential energy difference between differentiation state and the saddle point, 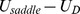. Here, 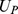 and 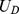 denotes respectively potential at the minimum for the stem cell attractor and the differentiation attractor, and 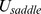 denotes the potential at the saddle point between these two basins of attractions. The results of barrier heights are based on RMSD method from Langevin dynamics (Figure S2). We projected the whole network landscape to two dimensions (RMSD1 and RMSD2). For this 2-dimensional landscape, we can acquire saddle points, local minimums and then barrier heights.

In this way, 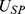 measures the global stability of the stem cell state or the stability of the differentiation process and 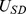 measures the stability of the differentiation state or the stability of the reprogramming process. When the system has larger 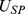 (stem cell state barrier or barrier for differentiation process 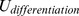) and smaller 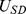 (differentiation state barrier or barrier for reprogramming process 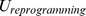), the stem cell state is more stable and the system is inclined to stay in the stem cell state. The differentiation process (transition from stem cell state to differentiation state) is hard to realize, because the system must go across a large barrier in order to escape from the stem cell state to differentiation state. The reprogramming process (the reverse process of differentiation, transition from differentiation state to stem cell state) is relatively easy to realize in this case (small 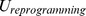). In contrast, if 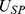 (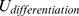) is small and 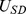 (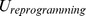) is larger for the stem cell system, it will be advantageous for differentiation process and difficult for reprogramming process, because the system only needs to overcome a small barrier to go from the stem cell state to the differentiation state (small 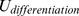), but a large barrier from differentiation state to pluripotent stem cell state (large 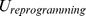).

**Figure 4:**
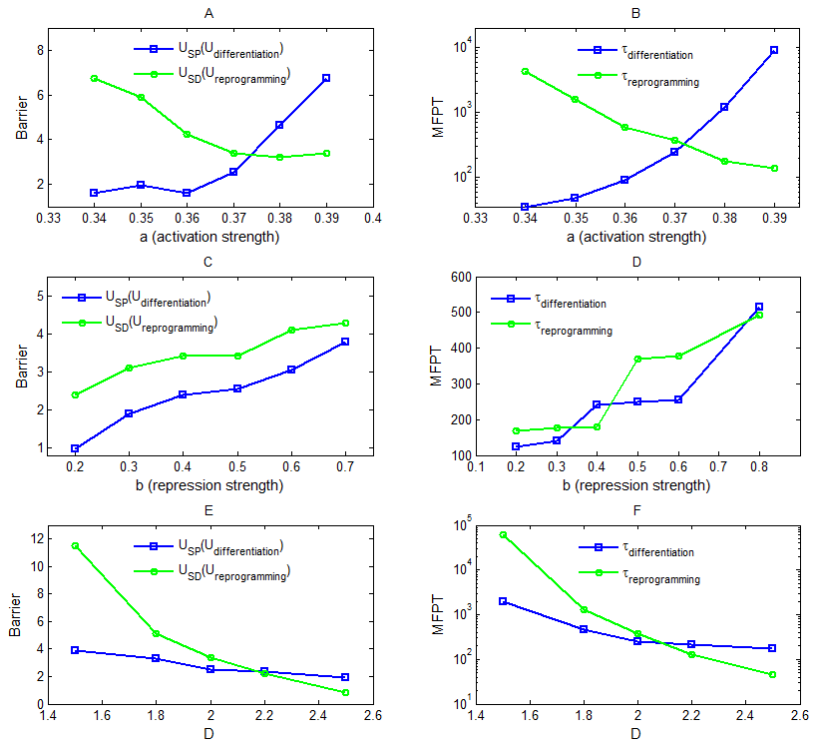
The barrier height and MFPT (mean first passage time) results when the activation strength 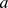, the repression strength 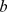 as well as the noise level 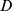 changes (Langevin dynamics). (A)(B) show that when 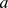 increases, stem cell state becomes more stable, the barrier for stem cell state 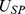 (or the barrier for differentiation process 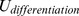) increases, and the MFPT for differentiation process from stem cell state to differentiation state (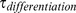) increases. By contrast, When 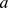 increases, differentiation state becomes less stable, the barrier for differentiation state 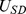 (or the barrier for reprogramming process 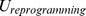) decreases, and the MFPT for reprogramming process from differentiation state to stem cell state (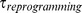) declines. (C)(D) show that when 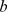 increases, the barrier for stem cell state 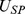 (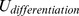), the barrier for differentiation state 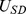 (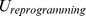), the MFPT for differentiation process (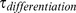), and the MFPT for reprogramming process (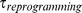) all increase. (E)(F) show that when noise level 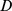 increases, the barrier for stem cell state 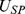 (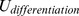), the barrier for differentiation state 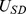 (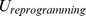), the MFPT for differentiation process (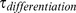), and the MFPT for reprogramming process (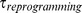) all decrease.

Figure 4 (A) shows respectively the barrier heights for differentiation process (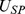 or 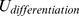) and the reprogramming process (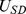 or 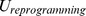) when activation strength 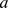 changes. We can see that with the activation constant 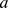 increased, 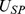 becomes larger and 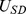 declines. It indicates that the enhancement of activation regulation in the network leads to more stable stem cell state, making it easier for the transition from the differentiation state to the stem cell state, and correspondingly the barrier height of differentiation process 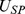 is raised. Meanwhile, when activation links are strengthened, the differentiation state becomes less stable, the system is not inclined to stay at differentiation state with a smaller barrier height of reprogramming process 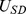 (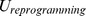). This implies that changing the strength of the activation links in the gene regulatory network provide a way to regulate the differentiation process or the reprogramming process and make the system inclined to differentiation or inclined to reprogramming determined by the relative stability of the two basin of attraction - stem cell attractor and differentiation attractor. The relative stability of these two attractors can be quantified by the landscape topography - that is, the barrier height. Indeed, some previous work have showed that changing self activation regulatory strength provide a possible mechanism for cell differentiation and reprogramming motifs involving two genes [11], [13], [14], [22].

We also investigated the kinetics or speed of differentiation and reprogramming according to the mean first passage time (MFPT), in order to further quantify the dynamics of differentiation and reprogramming process. We calculated the mean first passage time (MFPT) from the trajectory based on Langevin dynamics. In Figure S2B, left attractor represent ES state, the right attractor represents differentiation state, and this landscape is obtained by collecting the statistics through histogram or distribution of the temporal trajectories for 52 variables. Starting from a random initial state at ES attractor, following the temporal evolution trajectory of system, we can find the time where the system first enters into the differentiation attractor. The time difference between initial time and the final time is defined as the first passage time (FPT) for differentiation process. Repeating this process and obtaining the average of the FPT is defined as the MFPT for differentiation process. In the same way, we can obtain the MFPT for reprogramming.

MFPT reflects the average transition time of the system from one state to another state in the state space of gene regulatory networks, and therefore can be used to quantify the ability of a system switching from one state to another state. The results of MFPT are shown in Figure 4 (B). It can be seen that for the differentiation process, the MFPT of differentiation (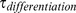, blue line) is longer when the activation strength 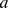 increases. This implies that when activation is larger the system need more time to jump from the stem cell state to the differentiation state. Cells have more chances to stay in the stem cell state, and therefore the increase of the activation strength is disadvantageous to the progress of differentiation. On the contrary, the decrease of activation makes MFPT of differentiation smaller, and thus cells are inclined to jump from stem cell state to differentiation state. The decrease of the activation strength represents the direction of the differentiation process. For the reprogramming process, the MFPT of the reprogramming 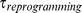 decreases as the activation strength increases, which means that when activation strength increases the system needs less time to jump from differentiation state to stem cell state, therefore making reprogramming easier.

We can find that the global barrier heights and the MFPT have the same trend for differentiation and reprogramming, and both of them can serve as the quantitative measure to global stability of the two attractors and kinetic speeds.

We also explored the influence of changing the repression strength 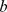 and the noise level 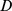 on the landscape topography. Figure 4(C,D) show the landscape results when the repression strength 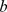 is changed. It shows that when 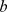 is increased, the barrier for differentiation process 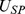 (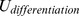), the barrier for reprogramming process 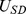 (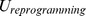), the MFPT for differentiation process from stem cell state to differentiation state (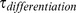), and the MFPT for reprogramming process from differentiation state to stem cell state (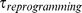) all increase. This might be because that the increase of mutual repression (a lot of the repression links are mutual repression between stem marker genes and differentiation marker genes) makes transitions between stem cell states and differentiation states harder, and thus the barriers and the MFPT both increase.

Figure 4(E,F) show the landscape results when the noise level 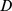 (diffusion coefficient in Langevin dynamics) is changed. It shows that when 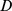 is increased, the barrier for differentiation process 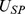 (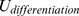), the barrier for reprogramming process 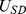 (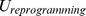), the MFPT for differentiation process from stem cell state to differentiation state (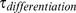), and the MFPT for reprogramming process from differentiation state to stem cell state (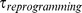) all go down. This can be explained that the increase of fluctuations makes both the stem cell state and the differentiation state less stable, and the transitions between the stem cell state and the differentiation state become easier, reflected by the decrease of the barriers and the MFPT. Meanwhile, in Figure 4(E,F) we can find that with 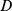 decreased the barrier for differentiation process 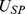 (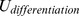) and the MFPT for differentiation (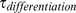) decline slower than the barrier for reprogramming process 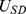 (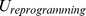) and the MFPT for reprogramming (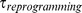). This shows that as the noise goes up the differentiation state becomes more stable relatively, which might provide a possibility for noise-induced differentiation or reprogramming [23], [24]. We need to stress that our non-equilibrium potential barrier 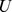 is dimensionless and directly related to the steady state probability 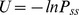 while the equilibrium potential barrier conventionally has a dimension as 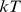 such that 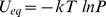. 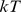 plays the role of the noise. This is why our non-equilibrium dimensionless 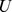 usually changes with noise while 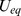 usually does not.

### Dynamical Transition Path for Differentiation and Reprogramming

We also used Langevin dynamics method to investigate the dynamics of the system (See methods for details), because it can provides the dynamical trajectory of the developmental system under fluctuating environments. Figure S2 show the landscape comparisons at different activation strength 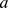, we can find that when 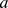 is large (Figure S2 (A), 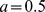) the stem cell state attractor is dominant, showing only one stable basin of attraction on the landscape graph, and when 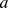 decreases to 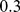, the differentiation state attractor is dominant (Figure S2 (D), 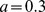). Specifically, when 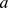 gradually decreases from 0.5 to 0.3, the stem cell state becomes less and less stable (differentiation barrier decreases and MFPT for differentiation decreases) and the differentiation state becomes more and more stable (reprogramming barrier increases and MFPT for reprogramming increases) until being dominant, demonstrating that the system of stem cell experiences a transition from stem cell state to differentiation state with activation strength 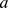 decreased. This implies that controlling the strengthes of the activation links between different marker genes might provide a mechanism for cell fate determination, differentiation or reprogramming. The regulation changes during the developmental processes are hinted in experiments by the effective regulations of the transcription factors mediated by Klf4 [25]. Therefore, we can see that the decrease of the activation strength represents the direction of development and differentiation. Along this direction, the Waddington landscape is downhill. This might provide a possible explanation for the direction (time arrow) for the developmental process hinted from the downhill trend of the Waddington landscape caused by the gene regulation changes. We suggest the direction or time arrow of the development by changing regulations leading to the underlying divergent funneled (with more states at the bottom in contrast to convergent funnel in protein folding) Waddington landscape is from natural selection. The regulation changes leading to the downhill Waddington landscapes for development are selected due to the emergence of the associated biological function (differentiation). The regulations not leading to the downhill Waddington landscape and failed for generating successful development and differentiation will not be selected and therefore become extinct in evolution.

To study the dynamics of developmental process when 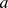 changes from 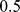 to 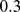, we specify activation constant 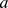 changing from large to small with time, 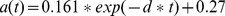. Here 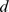 represents the rate of decreasing of 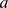 (parameters in this equation are selected in order to acquire a suitable dynamical transition trajectory from 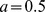 to 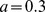). We assume that the activation strength 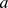 decreases in the developmental and differentiation process due to the regulations of the other genes in the network. Then we can obtain the trajectory of the stem cell system with the activation strength 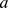 changed (RMSD as coordinates from Langevine dynamics).

**Figure 5:**
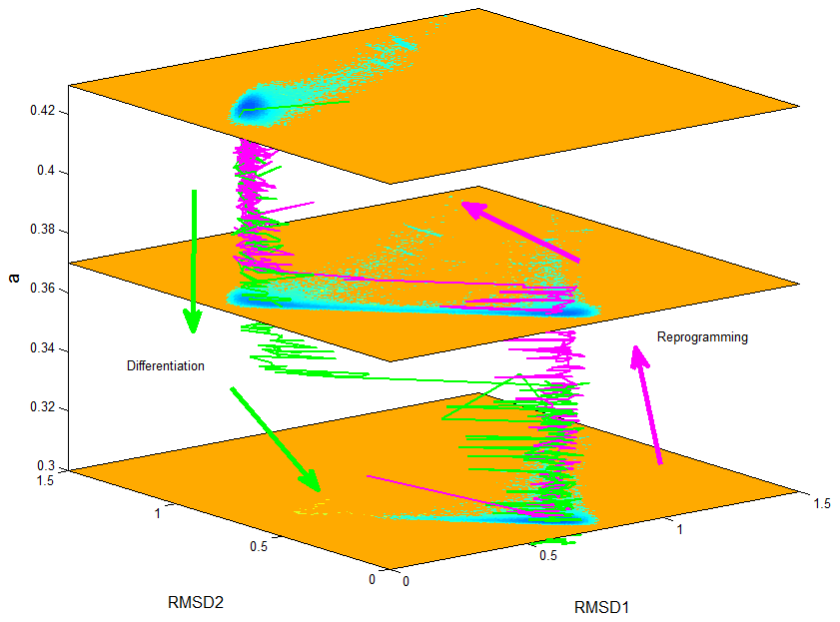
The differentiation and reprogramming trajectories on the landscape background. 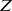
 axis represents activation strength 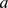. Three 2-dimensional landscape from up to down separately correspond to 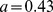, 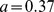 and 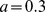. Green color represents differentiation trajectory (from stem cell state to differentiation state), and magenta color represents reprogramming trajectory (from differentiation state to stem cell state).

Figure 5 shows the dynamical transition path of the differentiation process (green line) and the reprogramming process (magenta line) on the underlying landscape. 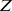 axis represents activation strength 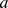. Three 2-dimensional landscape pictures represent the landscape of the stem cell network respectively for 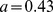, 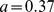, and 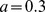. It can be seen clearly that as the differentiation progresses (represented by decrease of 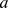), the landscape of the stem cell network changes gradually from a dominant stable stem cell attractor, to a balanced bistability, and finally to a dominant stable differentiation attractor. In the mean time, we can see that the two paths of differentiation and reprogramming are irreversible (green line and magenta line are not identical), which is consistent with the dominant path results from path integral. From transition path trajectories, we can see that for the development and differentiation process the trajectory first haunts around the stem cell state attractor, and then jump to the differentiation state attractor after 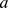 decreases to a critical value (we define it 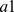 here). By contrast, for the reprogramming process, the trajectory firstly haunts around the differentiation state attractor, and jump to the stem cell attractor after 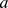 increases to a critical value 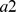. We notice that 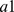 and 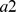 is not in the same place, which just effects the fact of irreversible transition paths. Indeed, 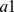 is smaller than 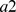, providing a hysteresis loop for the bistable switch. This result reflects one of the common characteristics for biological bistability: the existence of hysteresis for bistable switch, which comes from the feedback loops and provides an explanation of the irreversibility for the bistable switch. Figure 5 provides a quantified yet realistic Waddington landscape picture of differentiation and reprogramming.

As we did for the dominant path, we also monitored the differentiation and reprogramming kinetic paths with the activation strength 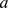 changed (separately shown in Table S6 and Table S7) in terms of certain key marker genes NANOG, GATA6, and CDX2. Similar to the analysis about dominant paths from path integrals, we can find that for the differentiation process the cell experiences an intermediate state (low NANOG/low GATA6/low CDX2 or low stem cell marker/low differentiation marker) along the path from the stem cell state to the differentiation state. For the reprogramming path, we can see that the cell also experiences an intermediate state (high NANOG/high GATA6/high CDX2, or high stem cell marker/high differentiation marker) along the path from the differentiation state to the stem cell state. These results have the consistent predictions with the dominant path analysis, which is that the cellular differentiation needs to experience an intermediate double low state (both stem cell marker genes and differentiation marker genes have low expression level), and the cellular reprogramming needs to experience an intermediate double high state (both stem cell marker genes and differentiation marker genes have high expression level). We expect that these predictions can be tested by experiments in the future, as well as help to design the differentiation and reprogramming strategies.

### Global Sensitivity Analysis for Repression and Activation Connections

We also did a global sensitivity analysis of parameters for the stem cell network in order to discover the key parameters or connections in the network affecting the stability and kinetic transitions of both the stem cell state and the differentiation state. Giving parameters, here representing the strength of 123 links in the stem cell network at a perturbation level 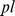, we can explore the influence of these parameters on the stability of the system by comparing the change of landscape topography quantified by the barrier heights.

We firstly exploited the self consistent approximation method [12], [19] to obtain those most important parameters - that is, by finding those parameters affecting barrier heights of the system critically. Specifically, we changed the value of each of the activation and repression constant 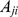 and 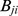 (Eq. (2), the parameters 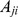 and 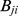 are only used for the global sensitivity analysis) by giving a percentage 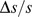 (here, 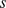 represents parameter 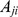 or 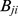, 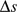 represents the change of parameter 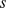, the value of 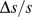 is controlled as between 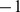 to 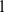) as the degree to change. Then for every mutation of parameters we compared the change of the landscape topography in terms of the barrier heights for both differentiation 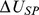 (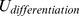) and reprogramming 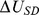 (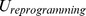). In this way, we acquired 20 most critical parameters or connections (14 of them are activation links and 6 of the others are repression links, see Text S1 and Figure S3 for details).

**Figure 6:**
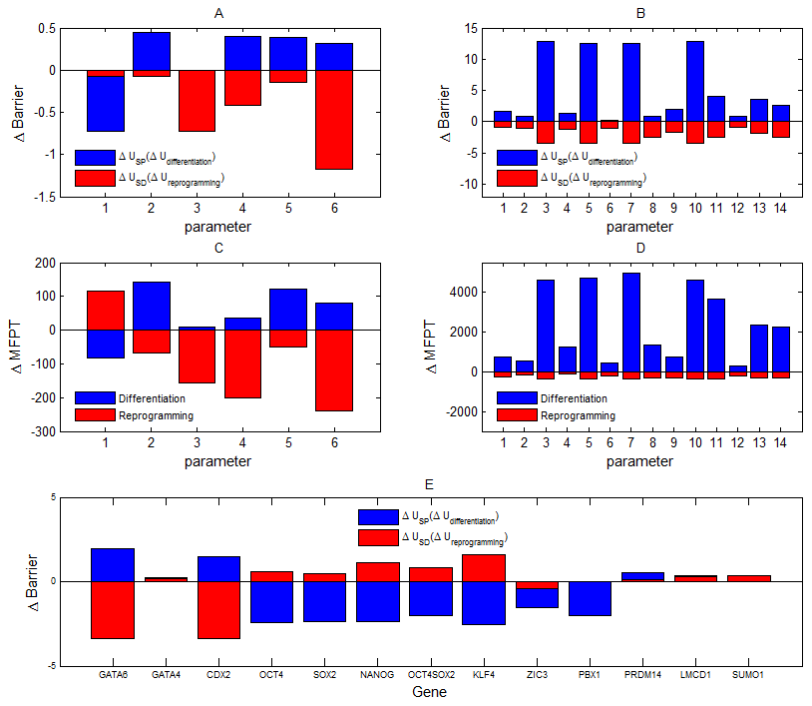
Results of the global sensitivity analysis in terms of barrier height and MFPT (mean first passage time) when parameters are changed. The results in (A) are for 6 repression links (named respectively as R1,R2,…,R6, see Table S2) based on the change of barrier heights (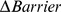). The results in (B) are for 14 activation links (named respectively as A1,A2,…,R14, see Table S3) based on barrier heights. Blue bars represent the change of 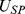 (barrier for differentiation process), red color represent the change of 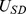 (barrier for reprogramming process). (C) and (D) separately show the corresponding results in terms of the change of MFPT (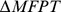). Blue bars represent the MFPT change for differentiation process, and red bars represent the MFPT change for reprogramming process. (E) shows the corresponding global sensitivity for the knockdown of individual genes.

In the following, we employed the Langevin dynamics to further obtain the change of barrier heights when these 20 parameters are changed, because by the Langevin dynamics the landscape of the system can be acquired directly by the statistics of the trajectories of the system - not through approximation. Figure 6 shows the results of the global sensitivity analysis for the 20 parameters or connections (see Text S1 for details). Figure 6 (A) shows the results for 6 repression links, and Figure 6(B) shows the results for 14 activation links. Blue bars represent the change of the barrier for stem cell state (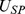), and the red bars represent the change of the barrier for differentiation state (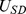).

In Figure 6 (A), x axis represent 6 parameters or connections. The 6 links are respectively corresponding to: 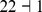 (link R1, 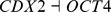), 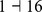 (link R2, 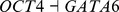), 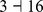 (link R3, 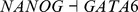), 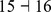 (link R4, 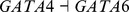), 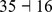 (link R5, 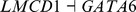), 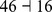 (link R6, 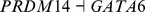). Here, 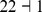 represents the repression regulation from gene CDX2 to gene OCT4 (see Text S1 for the detailed relation of the order numbers of genes and the corresponding genes). We can see that when the repression of CDX2 to OCT4 increases, 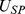 (stem cell state barrier) decreases significantly and 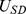 (differentiation state barrier) decreases slightly, making it easier to jump from stem cell state attractor to differentiation state attractor. Some experimental results have showed the importance of CDX2 to cell differentiation, which indicate that at the blastocyst stage Oct4 is gradually downregulated in the outer trophectoderm (TE, one cell type of differentiation state) cells by Cdx2 through direct physical interaction and transcriptional regulation [26]. In addition, repression link 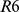 (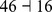 representing gene PRDM14 represses gene GATA6, here we defined a name for every repression and activation link, see Table S2 and Table S3 for the definition of link names) represents the repression of gene PRDM14 to gene GATA6. According to experimental results when PRDM14 is activated, the reprogramming is enhanced [27], which is also reflected in our global sensitivity analysis results that strengthening repression link 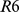 make 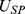 increase and 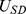 decrease.

For the repression link R2 (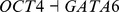), R3 (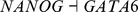), R4 (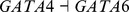), R5 (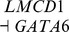), they are all the repression regulations from either stem cell marker genes (OCT4, NANOG) or other genes (GATA4, LMCD1) to the key differentiation marker gene 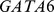. So, our global sensitivity analysis provide some predictions that increasing above 4 repression links will promote cellular reprogramming, since the increase of these 4 repression links make the stem cell barrier 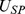 increase and differentiation barrier 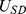 decrease. The repression of the differentiation marker gene 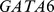 will strength the stem cell maker genes 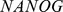 due to the repression of 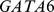 to 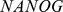, and then promote the cellular reprogramming. We found some experimental evidences that indicated the forced expression of Gata6 in embryonic stem (ES) cells is sufficient to induce the proper differentiation program [28]. This can provide some confirmation for the above 4 repression links, since these links are all the repression links to GATA6, and the global sensitivity analysis for these 4 links shows that increasing them will inhibit differentiation. Therefore, these predictions are reasonable and can be further validated by experiments. We can also see that among the 6 top important repression links, 5 of them are related to the repression of gene GATA6 (when these 5 repression links are strengthened, reprogramming becomes easier in that 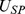 increases and 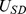 decreases), which should be due to the repression of 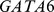 to stem cell marker gene 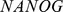.

Figure 6(B) shows the global sensitivity analysis results for 14 activation links, in which x axis represents separately: A1: A1:3→1(*NANOG→OCT*4);*A*2 : 4→1(*OCT*4*SOX*2→*OCT*4);*A*3 : 3→2(*NANOG→SOX*2);*A*4 : 4→2(*OCT*4*SOX*2→*SOX*2);*A*5 : 3→3(*NANOG→NANOG*);*A*6 : 4→3(*OCT*4*SOX*2→*NANOG*);*A*7 : 5→3(*KLF*4→*NANOG*);*A*8 : 7→3(*ZIC*3→*NANOG*);*A*9 : 11→3(*PBX*1→*NANOG*);*A*10 : 1→4(*OCT*4→*OCT*4*SOX*2);*A*11 : 2→4(*SOX*2→*OCT*4*SOX*2);*A*12 : 2→7(*SOX*2→*ZIC*3);*A*13 : 3→7(*NANOG→ZIC*3);*A*14 : 3→11(*NANOG→PBX*1) (arrows represent activation regulation, 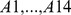 represent the name of the activation links, see supporting information for details). It can be seen that increasing the strength of the 14 activation connections all increase the barrier heights of stem cell state and decrease the barrier heights of the differentiation state, which means that the stem cell state becomes more stable and the differentiation state becomes less stable and it's easier for the system to make a transition from differentiation state to the stem cell state, i.e. increasing these activation links promote reprogramming progression of the system. We can see that among these 14 activation links, the first 11 of them are all activation links from other genes to the key stem cell marker genes (OCT4, SOX2, NANOG, OCT4SOX2). Undoubtedly, strengthening these 11 activation links will promote the cellular reprogramming process. Especially, among the 4 activation links which influence barrier greatly (link A3,A5,A7,A10, Figure 6(*B*)), two of them are activation links to NANOG, this shows activating NANOG is a robust way for reprogramming [29], [30]. For the last 3 activation links from global sensitivity analysis, we can see that they represent the activation of the key stem cell marker genes (NANOG, SOX2) to other stem cell marker genes (ZIC3, PBX1). The increase of these 3 activation links will increase the expression of 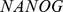, because of the activation of 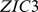 and 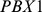 to 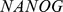, and thus promote the cellular reprogramming process. We also found some experimental evidences supporting our global sensitivity results described above. Experiments show that the increase of Klf4 promote reprogramming [25]. Additionally, among the 14 the 14 activation links, 5 of them are the activation regulation to NANOG. Some experiments show that Activation of Nanog can overcome barriers to lead reprogramming [31]. And some other links among the 14 activation links are activation links to OCT4 or SOX2. The experiments have confirmed that the activation of Oct4 and Sox2 can promote reprogramming [1]. Some recent recent experiments also indicate Zic3 can induce conversion to pluripotent stem cells [32].

Therefore, our global sensitivity analysis results are confirmed by some experiments. Our global sensitivity analysis results also provide the quantitative prediction about the effects of regulation links on the differentiation or reprogramming, which can be tested by further experiments. We need to emphasize that compared with the conventional sensitivity analysis which is usually local our sensitivity analysis is global since it is based on the global landscape topography quantified by the barrier height.

Additionally, we also quantified the global sensitivity of parameters through MFPT (mean first passage time), since MFPT reflects the average transition time from one basin of attraction to another, and therefore provides another quantitative measure for the stability of the system. Figure 6(C) and (D) show the influence of parameter change on the MFPT respectively for 6 repression links and 14 activation links. Comparing Figure 6(A) with (C), and (B) with (D), we can find that MFPT and barrier height give the consistent results on the global sensitivity analysis. Larger 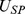 (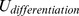) makes the transition from stem cell state to differentiation state harder, and thus means larger MFPT for differentiation. In contrast, larger 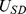 (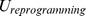) makes the transition from differentiation state to stem cell state harder, and thus larger MFPT for reprogramming. Therefore, 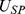 (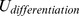) is corresponding to MFPT for differentiation, and 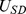 (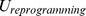) is corresponding to MFPT for reprogramming.

We also did the mutation for the knockdown of single nodes to see their influence to the landscape. Figure 6 (E) show the influence of knockdown of single genes on the barrier heights, (only showing the genes having large influence on barrier heights). The genes whose knockdown affect barrier heights critically include: GATA6,CDX2,OCT4,SOX2,NANOG,KLF4,ZIC3,PBX1. GATA6 and CDX2 are two key differentiation marker genes, so their knockdown promote reprogramming (increase barrier of stem cell state and decrease barrier of differentiation state). OCT4,SOX2,NANOG,KLF4,ZIC3,PBX1 are key stem cell marker, and it's reasonable that their knockdown promote differentiation (increase barrier of differentiation state and decrease barrier of stem cell state). These key gene markers have been highlighted in Figure 1.

**Figure 7:**
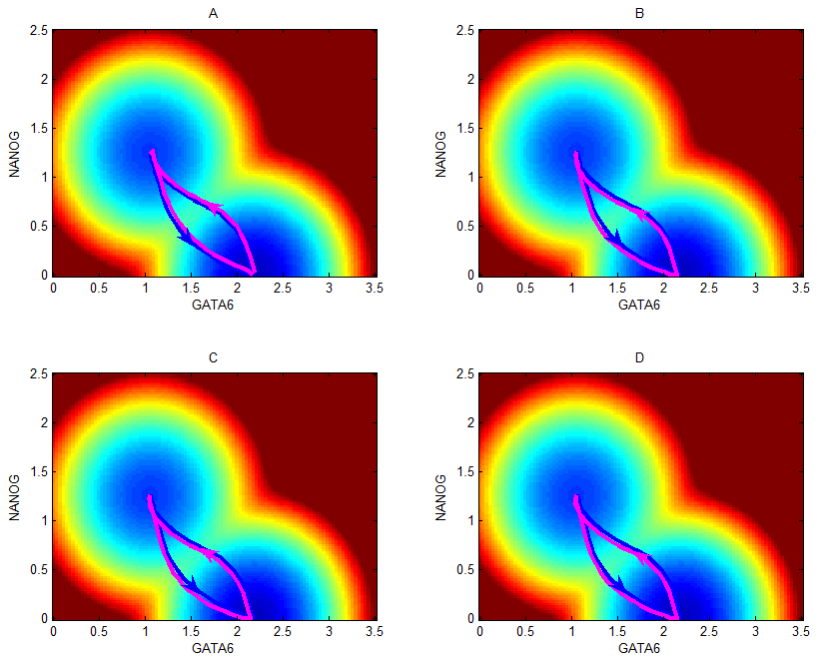
Global sensitivity for kinetic paths. The developmental and reprogramming paths comparisons before and after mutations are shown separately for mutation 1 (increase link R1, i.e. the repression of CDX2 to OCT4)(A), mutation 2(increase link R2, i.e. the repression of OCT4 to GATA6)(B), mutation 3 (increase link R3, i.e. the repression of NANOG to GATA6)(C), and mutation 4 (increase link R4, i.e. the repression of GATA4 to GATA6)(D). The blue paths represent kinetic paths before mutations, and the magenta paths represent the kinetic paths after mutations. The blue arrows represent differentiation direction (from left stem cell attractor to the right differentiation attractor), and the magenta arrows represent reprogramming direction (from right attractor to left attractor). The diffusion coefficient 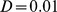.

We also did some mutations to see their effects on kinetic path based on path integral approach. Figure 7 show the influence of parameters on the kinetic paths separately for mutation 1 (increase the repression of CDX2 to OCT4), mutation 2 (increase the repression of OCT4 to GATA6), mutation 3 (increase the repression of NANOG to GATA6), mutation 4 (increase the repression of GATA4 to GATA6). The left attractor represent the stem cell state, and the right one represents the differentiation state. The blue paths are before mutations, the magenta paths are the results after mutations. The global sensitivity analysis (Figure 6(A)(C)) of mutation 2 (increase the repression of OCT4 to GATA6) show that mutation 2 can make stem cell state (left attractor in landscape) more stable, and differentiation state less stable, or the MFPT for differentiation become longer. While by comparison of paths in Figure 7 (B), the differentiation path (path from left attractor to right attractor) becomes more deviating (change from blue path to magenta path) from the shortest path, or the differentiation path become longer (so spending more time or MFPT increases), and the opposite results are for the reprogramming path. This is consistent with the barrier height and MFPT results in Figure 4(A)(C). In the same way, the path sensitivity for mutation 3, and mutation 4 (Figure 7(C)(D)) also show the differentiation path becomes more deviating (change from blue path to magenta path) from the shortest path, and thus spend more time, which are also consistent with the global sensitivity analysis results in terms of barrier height and MFPT in Figure 4(A)(C). In addition, for mutation 1 (Figure 7(A)), the path comparison shows that after mutation the differentiation path becomes closer to the shortest path, meaning that the differentiation process become easier. This is also consistent with the barrier and MFPT sensitivity for mutation 1, which shows that the differentiation state become more stable, i.e. this mutation promotes the differentiation process.

In summary, the global sensitivity analysis in terms of barrier, MFPT, and kinetic path provide a way to uncover the key factors critically determining the process of cellular differentiation and reprogramming (highlighted in black solid links in Figure 1). Some of our predictions are consistent with the experimental evidences. More importantly, we provided certain predictions about which regulation links in the stem cell network are critical to differentiation or reprogramming (Figure 6), which can be directly validated from relevant experiments in terms of both MFPT or the differentiation and reprogramming pathway.

### Conclusions

We uncovered the landscape of a stem cell developmental and differentiation network. Landscape shows that the stem cell gene regulatory network has two stable basins of attractions at specific parameter regions, one of which represents the pluripotent stem cell state and the other of which represents the differentiation state. In terms of the path integral approach, we acquired the kinetic paths for both development and reprogramming. Both landscape and curl flux determine the dynamics of the stem cell network. Flux leads the kinetic paths of the system deviating from the steepest descent path from gradient of potential, and the differentiation path and the reprogramming path are irreversible. Barrier heights based on landscape topography provide quantitative measures for the stability and kinetic transition of the two attractors. MFPT provide an avenue to acquire the information of transition rate or kinetic speed for the system to jump from an attractor to another. By the global sensitivity analysis in terms of barrier heights, MFPT, and kinetic path, we provided some predictions about the key genes and connections affecting differentiation and reprogramming significantly, which can be tested by experiments. Importantly, the key links and genes from global sensitivity analysis and biological paths we acquired can be used to guide the differentiation designs or reprogramming tactics.

The current stem cell network we employed only provides some general biological markers and their interaction about stem cell differentiation and reprogramming. With more biological details added into the stem cell network, such as a network including certain differentiation marker genes representing some different differentiation states [33], it can be anticipated that we can explore the landscape and paths not only for the differentiation or reprogramming process, but also for transdifferentiation process (the transition between different differentiation state cell types). In addition, in our current model, we only simulated the single cell behavior, not considering the effects of cell division. We hope that we can absorb the cell division to the model, since the cell division rate can influence the stem cell or differentiation cell populations [34].

Our approach provides a general way to investigate the global properties—landscape topography, transition rate, kinetic path—of large gene regulatory networks which only have information on interaction directions (activation or repression) without interaction strength. In particular, we provide a approach to investigate biological paths of high dimensional systems. Our approach can be applied to other gene regulatory networks or protein networks.

## Methods

### Model for the Stem Cell Developmental Network

A human stem cell network has been constructed by searching for literatures, which includes most of the main regulations in human embryonic stem cell (hESCs) as shown in Figure 1 [18]. This network includes 52 protein nodes (Table S1) and their interactions (total 123 links including 84 activation links and 39 repression links), in which red arrows represent activation and blue bars represent repression. There are 11 marker genes for Pluripotency state (iPS state or stem cell state) and 11 marker genes for differentiation state, which are separately colored in purple and cyan. The orange nodes represent genes that are activated by iPS marker genes, and the light red color nodes denote other genes.

The iPS marker genes include OCT4, SOX2, NANOG, Oct4-Sox2, KLF4, FOXD3, ZIC3, ZFP42, GDF3, TDGF1, PBX1, and the differentiation marker include FOXA2, AFP, SOX17, GATA4, GATA6, T, GATA2, GATA3, hCGa, hCGb, CDX2. Basically, the dynamics of the network is determined by the mutual repression of major ES marker genes (NANOG, OCT4, SOX2) and major differentiation marker genes (GATA4, CDX2). When the ES markers are highly expressed, the system will be in ES state, and when the differentiation markers are highly expressed, the system will be in differentiation state. Specifically, the trophectoderm lineage is determined by the antagonism between Oct4 and Cdx2 (mutual repression links in the network), whereas the mutually repressions between Gata6 and NANOG determine the primitive endoderm lineage [30], [35], [36]. Some other regulations of the network include the self-activation of some key marker genes (NANOG, GATA6, CDX2), as well as the mutual activation between ES marker genes. So, the bistable regulatory dynamics for the full 52 gene network is mostly determined by the antagonism between Oct4 and Cdx2, and the antagonism between GATA6 and NANOG. These major regulation links are for embryo stem cells [30], [35], [36].

These marker genes constitute a major stem cell gene regulatory network, which orchestrates some important cellular functions, such as the cell differentiation and reprogramming. For instance, transcription factors OCT4, SOX2 and NANOG play important roles to the early development of cell and propagation of undifferentiated embryonic stem cell [36], [37]. The protein OCT4 and the protein FOXD3 are transcriptional regulators expressed in embryonic stem cells. Down regulation of OCT4 is an essential requirement during gastrulation for proper endoderm development [38].

For the 52 node network, we constructed 52 corresponding ordinary differential equations describing dynamics of the system, in terms of Hill function representing their activation or repression interactions. The equation has the form as:
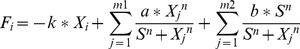
(1)


Here in Eq (1), i = 1,2,…,52, so totally there are 52 equations. 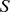 represents the threshold (inflection point) of the explicitly sigmoidal functions, i.e., the strength of the regulatory interaction, and 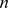 is the Hill coefficient which determines the steepness of the sigmoidal function [22]. Here, parameters for Hill function are specified as: 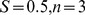. In addition, 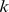 is self-degradation constant, 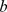 is repression constant, and 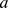 is activation constant. In the above equation, the first term represents self-degradation, the second term represents activation from node 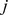 to node 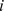 (m1 represents the number of activations to node 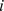, and this term represents self-activation when 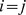), and the last item denotes repression from node 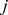 to node 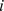 (m2 represents the number of repressions to node 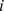, and this term represents self-repression when 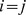). Here firstly we designated parameters value uniformly (Eq. (1)), i.e. all activation strength 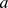 is same, and also for the repression strength 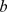, because so far we have no access for the information about the regulation strength — or the magnitude of activation and repression parameters — between different genes in the stem cell network.

In the global sensitivity analysis section (throughout the paper we use Eq(1) as the driving force, and the Eq (2) is only used in global sensitivity analysis section), we will change each specific activation strength 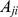 (representing the activation constant for the regulation from node j to node i) and repression strength 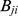 (representing the repression constant for the regulation from node j to node i) (Eq. (2)) to see their influence on the dynamics of the system. Throughout the paper, we use 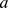 and 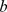 to separately denote uniform activation constant and repression constant. The parameters 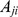 and 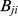 (Eq (2)) are only used in global sensitivity analysis section. The default parameter values (Figure 2) are set as: 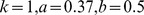.
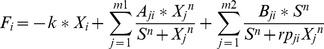
(2)


About the value of parameters, we choose parameter values according to the following criteria:

1. We chose parameter values according to some previous work [11], [13], [22]. In the work of Huang et [22], they explored a two gene system, where the region for producing bistability is given as: threshold S = 0.5–1.5, Hill coefficient n = 4–8.
2. For the degradation, activation and repression strength, we set them uniformly for different variables and set them in the same magnitude, because so far for stem cell network there is no such information about regulation strength which should come from the detailed biochemistry reactions involved in cellular developmental system. This method of choosing parameters has been used by some other works [11], [13], [22].
3. We chose those parameters that can satisfy some biological constrains, including producing steady state solution as well as producing bistability, since our purpose is to explore the cellular differentiation and reprogramming dynamics (two stable states). The random parameter selection does not satisfy these biological constraints.
4. In barrier height and MFPT section (changing activation strength a and repression strength b), as well as in global sensitivity analysis section, we did the perturbation for both activation strength and repression strength, which show the relative robustness of current parameter regions. We also provided the results of different parameters for different genes.

### Self Consistent Mean Field Approximation

The time evolution the dynamical systems are governed by the diffusion equations. Given the system state 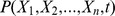, where 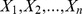 is the concentration or populations of molecules or species, we expected to have N-coupled differential equations, which are difficult to solve. Following a self consistent mean field approach [8], [12], [19], we split the probability into the products of individual ones: 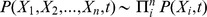 and solve the probability self-consistently. This effectively reduces the dimensionality from 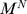 to 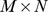, and thus makes the problem computationally tractable.

However, for the multi-dimensional system, it is still hard to solve diffusion equations directly. We can start from moment equations and then simply assume specific probability distribution based on physical argument, meaning that we give some specific connections between moments. In principle, once we know all moments, we can construct the probability distribution. For example, Poisson distribution has only one parameter, so we may calculate all other moments from the first moment, that is the mean. Here we use gaussian distribution as approximation, then we need two moments, mean and variance.

When diffusion coefficient 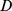 is small, the moment equations can be approximated to [39], [40]:
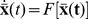
(3)

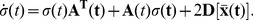
(4)


Here, 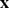, 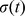 and 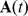 are vectors and tensors, and 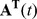 is the transpose of 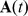. The matrix elements of A is 
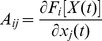
. In terms of this equation, we can solve 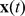 and 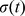. Here, we consider only diagonal elements of 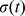 from mean field splitting approximation. Therefore, the evolution of probabilistic distribution for each variable could be acquired using the mean and variance based on gaussian approximation (see Text S1 for detailed deduction process of Gaussian Approximation method):
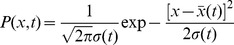
(5)


The probability obtained above corresponds to one fixed point or basin of attraction. If the system allows multistability, then there are several probability distributions localized at every basin of attraction, but with different variations. Therefore, the total probability is the weighted sum of all these probability distributions. The weighting factors 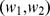 are the size of the basin, representing the relative size of different basin of attraction. For example, for a bistable system, the probability distribution takes the form: 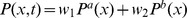, here 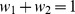. Here, we determine the weights 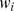 by giving a large number of random initial conditions for ODEs to find solution, and then collect the statistics for different solution. For example, for a bistable system, if 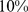 initial condition goes to the first steady state, and 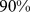 initial condition goes to the second steady state, then the weight 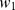 for the first basin is 0.1 and 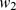 for the second basin is 0.9. The multistability comes from the solution of 52 ODEs giving a large number (100000) of random initial values. We give large number of random different initial conditions for ODEs for solution at a fixed parameter set. By collecting the statistics of the solution, we can determine if the system is monostable or bistable or mutistable at current parameter region. In our current work, for 52 dimensional system, we can acquire 52 dimensional probability distribution. To exhibit the results in a 2-dimensional space, we integrated out the other 50 variables and left two variables NANOG and GATA6.

Finally, once we have the total probability, we can construct the potential landscape by the relationship with the steady state probability: 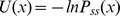. In the gene regulatory network system, every parameter or link contributes to the structure and dynamics of the system, which is encoded in the total probability distribution, or the underlying potential landscape.

For nonequilibrium gene regulatory systems, the driving force 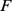 can not be written as the gradient of potential 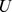, like the equilibrium case. In general, 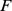 can be decomposed into a gradient of the potential and a curl flux force linking the steady state flux 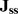 and the steady state probability 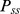 [10], [12] (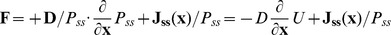). 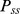 denotes steady state probability and potential U is defined as 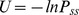. The probability flux vector 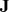 of the system in concentration or gene expression level space 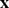 is defined as [32]: 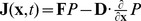.

In the 52-dimensional protein concentration space, it's hard to visualize 52-dimensional probabilistic flux. Approximately, we explored the associated 2-dimensional projection of flux vector: 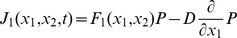 and 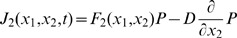.

In addition, to validate the Gaussian approximation method, we provided the landscape results from Gaussian distribution approximation of the 2-dimensional case for GATA1/PU1 [11], [13], and made comparisons for this 2-dimension case between Gaussian approximation method and Langevin dynamics method (Figure S4). We can see that the landscapes from Gaussian approximation preserve the similar global properties (the number of attractors, the relative stability of basin of attractions) as the Langevin dynamics method.

### Paths for Differentiation and Reprogramming from Discretized Dynamics

The landscape in Figure 2 only is the 2-dimensional projection of the whole 52 dimensional state space. In order to demonstrate the cell states and the transitions between different cell types in the complete state space, we projected the expression level of the 52 gene variables to binary states, and acquired discretized dynamics results of the network (Figure 3).

We first used the Langevin dynamics to obtain the stochastic dimensionless trajectories of the 52 dimensional system. Then the trajectory is converted to discrete trajectories by setting the value ((maximum value - minimum value)/2+minimum value) of every variable as the cutoff (cutoff is chosen so that two up/down states are well separated), i.e. the value higher than the cutoff is set to 1 (indicating high expression), while the value lower than the cutoff is set to 0 (indicating low expression). So, we can obtain the discrete trajectories for 52 variables of the system. For a 52 dimension system, there will be 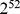 states even in discrete case (every variable has two value, 1 represent high expression, 0 represent low expression), which cannot be handled computationally. So, we chose the major 22 marker genes to present the discrete system, which has 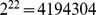 states. For example, the stem cell state is represented by the binary number 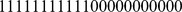 (representing expression level from gene 1 to gene 22, 1 for high expression, 0 for low expression), and for the differentiation state, it is represented by 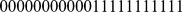. By the statistics for the discrete trajectory, we can obtain the appearing probability separately for 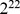 different states. To present the results, we set a probability cutoff 0.0002 (only states with higher probability than 0.0002 are chosen, the cutoff is chosen so that the major states can be presented in a figure, not too many or too few states, i.e. we only demonstrate the states and paths with higher probability). Figure 3 shows the differentiation and reprogramming process represented by 313 cell states (nodes) and 329 transition jumps (edges) between the different cell states. We believe that these 313 states with higher probability can capture the major states and regulation dynamics of the system. The sizes of nodes and edges are separately proportional to the occurrence probability of the corresponding states and paths. Red nodes represent states which are closer to stem cell states, and blue nodes represent states which are closer to differentiation states. Especially, we acquired the dominant kinetic paths as the biological paths from path integral formulism (see Path Integral section for detailed methods), which are shown as green and magenta paths (Figure 3) separately for differentiation and reprogramming process.

### Kinetic Path from Path Integral

In the cell, there exist external noise and intrinsic noise, which can be significant to the dynamics of the system [41], [42]. Therefore, a network of chemical reactions in noisy fluctuating environments can be addressed by: 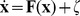. Here, 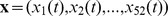 represents the vector of protein concentration or gene expression level. 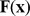 is the vector for the driving force of chemical reaction. 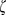 is Gaussian noise term whose autocorrelation function is 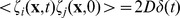, and 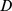 is diffusion coefficient matrix.

The dynamics for the probability of starting from initial configuration 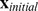 at t = 0 and ending at the final configuration 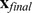 at time t, in terms of the Onsager-Machlup functional, can be formulated [13], [43] as: 
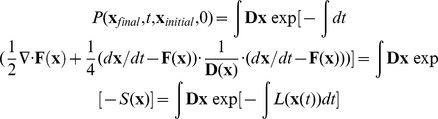
.


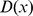
 is the diffusion coefficient matrix. The integral over 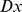 denotes the sum over all possible paths from the state 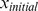 at time 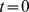 to 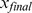 at time 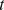. The exponent factor gives the weight of each path. Therefore, the probability of network dynamics from initial state 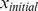 to the final state 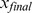 is equal to the sum of all possible paths with different weights. The 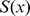 is the action and 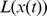 is the Lagrangian or the weight for each path.

The path integrals can be approximated with a set of dominant paths, since each path is exponentially weighted, and the other subleading path contributions are often small and can be neglected. Therefore, the dominant path with the optimal weights can be acquired through minimization of the action or Lagrangian. In our case, we identify the optimal paths as the biological paths, i.e. differentiation and reprogramming paths.

### Langevin Dynamics Method

A network of chemical reactions in noisy fluctuating environments can be addressed by 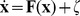. Here, 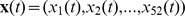 represents the vector of protein concentration. 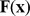 is the vector for the driving force of chemical reaction. In the cell, there exist external noise and intrinsic noise, which can be significant to the dynamics of the system [42], so the noise term 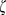 is added to force item for which Gaussian distribution is assumed, since the force 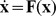 depict only the averaged dynamics of the system. The noise item is satisfied with: 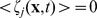 and 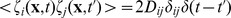
(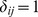 for 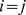, and 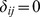 for 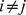). Here 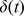 is the Dirac delta function, and 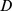 is diffusion coefficient matrix. The noise term is associated with the intensity of cellular fluctuations either from the environmental external fluctuations or intrinsic fluctuations. Under large 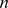 expansions, the process follows Brownian dynamics.

Following the Brownian dynamical trajectories with multiple different initial conditions by solving the above SDE (stochastic differential equations) iteratively, we can obtain the steady state distribution function 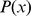 for the state variable 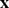 (relative gene expression value in the gene regulatory network), which is relevant to the potential energy function 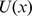 as 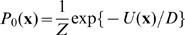. Here the partition function 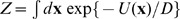. In this way, we acquire the potential energy landscape.

## Supporting Information

Figure S1 A flowchart for methods employed. GRN represents the gene regulatory network.
(EPS)

Figure S2Landscape change when activation strength 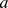 increase. (A) 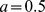, (B) 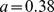, (C)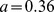, (D) 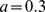. We can see as the activation strength 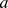 decreases the bistable landscape experience a transition from stem cell state (left attractor) to differentiation state (right attractor). The diffusion coefficient 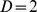. For 52 dimensional system, for visualization, we harnessed 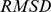 (root mean squared distance) as the coordinate to reduce the dimensionality to 2 dimension (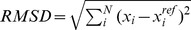, 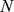 is the number of variables, and 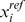 is the reference state, here we chose two potential minima as the reference states). 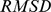 represents the distance between a state point and reference point in state space. In this way, from 52-dimensional trajectory, we can generate two new coordinates 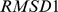 and 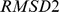, separately representing the distance from a state point to the reference state 1 (the potential minimum of stem cell attractor) and the reference state 2 (the potential minimum of differentiation state attractor).(PDF)

Figure S3Using self consistent approximation method to get barrier changes when parameters are changed. The results in A are for 84 activation links, and The results in B are for 39 activation links. Blue stairs represent the barrier for stem cell state 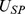 for different mutations, red stairs represent the barrier for differentiation state 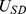 for different mutations. X axis in A represent all 84 activation links, and X axis in B represent all 39 repression links.(EPS)

Figure S4 Comparisons of self consistent approximation method and Langevin dynamics for a 2 gene model. (A) shows the network structure of 2 gene model (GATA1/PU1). (B) shows the comparisons of landscape using self consistent approximation method (first row) and Langevin dynamics method (second row). Parameters are set: D = 0.05 (diffusion coefficient), k = 1 (degradation), b = 1 (repression), S = 0.5, n = 4, and a (activation) is changed from left to right (1.2, 1, 0.2).
(PDF)

Table S1 Names of 52 gene in the stem cell network and the corresponding order number.
(PDF)

Table S2 Repression link names in the sensitivity analysis and the corresponding regulations they represent. The order numbers for causal and target genes are shown, which are corresponding to the gene name in Table S1.
(PDF)

Table S3 Activation link names in sensitivity analysis and the corresponding regulations they represent. The order numbers for causal and target genes are shown, which are corresponding to the gene name in Table S1.
(PDF)

Table S4 Differentiation path characterized by high/low expression level of 22 marker genes in Figure 3 of main text. The line of gene ID represent the corresponding genes in Table S1. From the line stem cell to line differentiation, every line represents a cellular states. Stem cell represent the stem cell states, and differentiation represents differentiation states. 1 denotes high expression level, and 0 represents low expression level.
(PDF)

Table S5 Reprogramming path characterized by high/low expression level of 22 marker genes in Figure 3 of main text. The line of gene ID represent the corresponding genes in Table S1. From the line differentiation to line stem cell, every line represents a cellular states. Stem cell represent the stem cell states, and differentiation represents differentiation states. 1 denotes high expression level, and 0 represents low expression level.
(PDF)

Table S6Differentiation path as activation strength 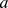 changed characterized by high/low expression level of 22 marker genes in Figure 5 of main text. The line of gene ID represent the corresponding genes in Table S1. From the line differentiation to line stem cell, every line represents a cellular states. Stem cell represent the stem cell states, and differentiation represents differentiation states. Intermediate represents the intermediate double low state (both stem cell marker and differentiation marker genes have low expression level). 1 denotes high expression level, and 0 represents low expression level.(PDF)

Table S7Reprogramming path as the activation strength 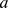 changed characterized by high/low expression level of 22 marker genes in Figure 3 of main text. The line of gene ID represent the corresponding genes in Table S1. From the line differentiation to line stem cell, every line represents a cellular states. Stem cell represent the stem cell states, and differentiation represents differentiation states. Intermediate represents the intermediate double high state (both stem cell marker and differentiation marker genes have high expression level). 1 denotes high expression level, and 0 represents low expression level.(PDF)

Text S1 Supplementary results and methods.
(PDF)
