# Supplementary figures and images for: Quantifying Cell Fate Decisions for Differentiation and Reprogramming of a Human Stem Cell Network: Landscape and Biological Paths
Source: PLoS Comput Biol. 2013 Aug 1;9(8):e1003165. doi: 10.1371/journal.pcbi.1003165 (PMC3731225; doi:10.1371/journal.pcbi.1003165)

A

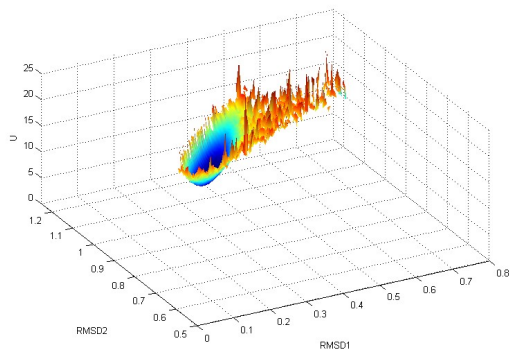

B

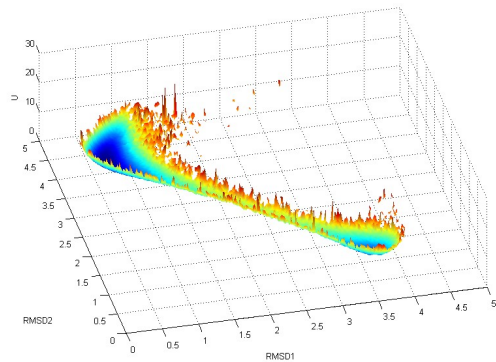

C

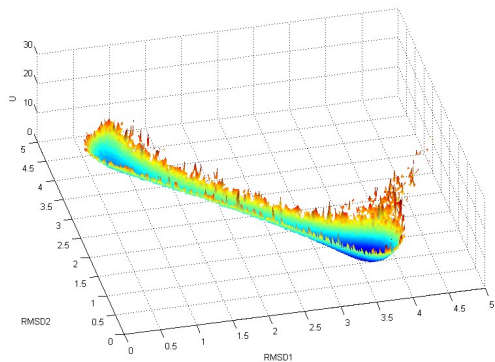

D

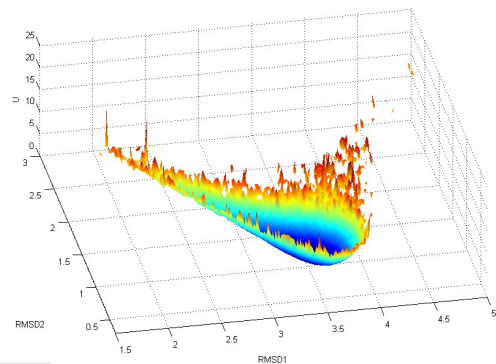

Supplement: Figure S2 — Landscape change when activation strength increase. (A) , (B) , (C), (D) . We can see as the activation strength decreases the bistable landscape experience a transition from stem cell state (left attractor) to differentiation state (right attractor). The diffusion coefficient . For 52 dimensional system, for visualization, we harnessed (root mean squared distance) as the coordinate to reduce the dimensionality to 2 dimension (, is the number of variables, and is the reference state, here we chose two potential minima as the reference states). represents the distance between a state point and reference point in state space. In this way, from 52-dimensional trajectory, we can generate two new coordinates and , separately representing the distance from a state point to the reference state 1 (the potential minimum of stem cell attractor) and the reference state 2 (the potential minimum of differentiation state attractor). (PDF) [file pcbi.1003165.s002.pdf]

A

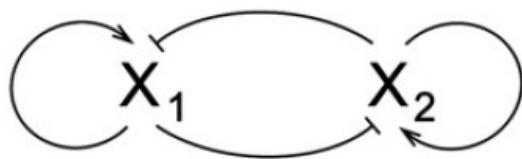

B

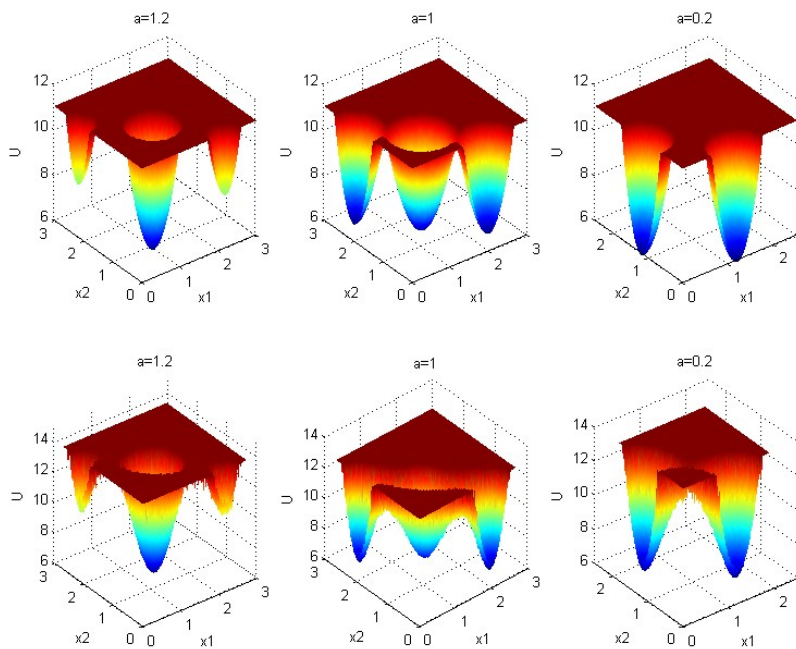

Supplement: Figure S4 — Comparisons of self consistent approximation method and Langevin dynamics for a 2 gene model. (A) shows the network structure of 2 gene model (GATA1/PU1). (B) shows the comparisons of landscape using self consistent approximation method (first row) and Langevin dynamics method (second row). Parameters are set: D = 0.05 (diffusion coefficient), k = 1 (degradation), b = 1 (repression), S = 0.5, n = 4, and a (activation) is changed from left to right (1.2, 1, 0.2). (PDF) [file pcbi.1003165.s004.pdf]
