# Supplementary material for: Quantifying Cell Fate Decisions for Differentiation and Reprogramming of a Human Stem Cell Network: Landscape and Biological Paths
Source: PLoS Comput Biol. 2013 Aug 1;9(8):e1003165. doi: 10.1371/journal.pcbi.1003165 (PMC3731225; doi:10.1371/journal.pcbi.1003165)
Supplement: Table S1 — Names of 52 gene in the stem cell network and the corresponding order number. (PDF) [file pcbi.1003165.s005.pdf]

Table.S 1. Names of 52 gene in the stem cell network and the corresponding order number.

OCT4=1	SOX2=2	NANOG=3	OCT4Sox2=4	KLF4=5;	FOXD3=6;
ZIC3=7	ZFP42=8	GDF3=9	TDGF1=10	PBX1=11	FOXA2=12;
AFP=13	SOX17=14;	GATA4=15;	GATA6=16	T=17;	GATA2=18;
GATA3=19	hCGa=20	hCGb=21	CDX2=22	bCAT=23	BMP2=24
BMP2K=25	cAMP=26	HNF4A=27	CEBP=28	CSH1=29	E2F4=30
FOXA1=31	FOXO1A=32	LEF1=33	LEF1bCat=34	LMCD1=35	Mad =36
MadMax=37	MAX=38	MYC=39	MycMax=40	MYCSP1=41	NFYA=42
OCT4FOXD3=43	PIAS1=44	PIASy=45	PRDM14=46	SALL4=47	SP1=48
SP3=49	SUMO1=50	TCF3=51	ZNF206=52		

Number 1-11 of the genes are stem cell marker genes, and Number 12-22 of the genes are differentiation marker genes.
