# Supplementary material for: Quantifying Cell Fate Decisions for Differentiation and Reprogramming of a Human Stem Cell Network: Landscape and Biological Paths
Source: PLoS Comput Biol. 2013 Aug 1;9(8):e1003165. doi: 10.1371/journal.pcbi.1003165 (PMC3731225; doi:10.1371/journal.pcbi.1003165)
Supplement: Table S2 — Repression link names in the sensitivity analysis and the corresponding regulations they represent. The order numbers for causal and target genes are shown, which are corresponding to the gene name in Table S1. (PDF) [file pcbi.1003165.s006.pdf]

Table.S 2. Repression link names in the sensitivity analysis and the corresponding regulations they represent. The order numbers for causal and target genes are shown, which are corresponding to the gene name in Table S1.

Link Name	Causal Genes	Target Genes
R1	22	1
R2	1	16
R3	3	16
R4	15	16
R5	35	16
R6	46	16
