# Supplementary material for: Quantifying Cell Fate Decisions for Differentiation and Reprogramming of a Human Stem Cell Network: Landscape and Biological Paths
Source: PLoS Comput Biol. 2013 Aug 1;9(8):e1003165. doi: 10.1371/journal.pcbi.1003165 (PMC3731225; doi:10.1371/journal.pcbi.1003165)
Supplement: Table S3 — Activation link names in sensitivity analysis and the corresponding regulations they represent. The order numbers for causal and target genes are shown, which are corresponding to the gene name in Table S1. (PDF) [file pcbi.1003165.s007.pdf]

Table.S 3. Activation link names in sensitivity analysis and the corresponding regulations they represent. The order numbers for causal and target genes are shown, which are corresponding to the gene name in Table S1.

Link Name	Causal Genes	Target Genes
A1	3	1
A2	4	1
A3	3	2
A4	4	2
A5	3	3
A6	4	3
A7	5	3
A8	7	3
A9	11	3
A10	1	4
A11	2	4
A12	2	7
A13	3	7
A14	3	11
