# Supplementary material for: Quantifying Cell Fate Decisions for Differentiation and Reprogramming of a Human Stem Cell Network: Landscape and Biological Paths
Source: PLoS Comput Biol. 2013 Aug 1;9(8):e1003165. doi: 10.1371/journal.pcbi.1003165 (PMC3731225; doi:10.1371/journal.pcbi.1003165)
Supplement: Table S5 — Reprogramming path characterized by high/low expression level of 22 marker genes in Figure 3 of main text. The line of gene ID represent the corresponding genes in Table S1. From the line differentiation to line stem cell, every line represents a cellular states. Stem cell represent the stem cell states, and differentiation represents differentiation states. 1 denotes high expression level, and 0 represents low expression level. (PDF) [file pcbi.1003165.s009.pdf]

Table.S 5. Reprogramming path characterized by high/low expression level of 22 marker genes in Figure 3 of main text. The line of gene ID represent the corresponding genes in Table S1. From the line differentiation to line stem cell, every line represents a cellular states. Stem cell represent the stem cell states, and differentiation represents differentiation states. 1 denotes high expression level, and 0 represents low expression level.

gene ID	1	2	3	4	5	6	7	8	9	10	11	12	13	14	15	16	17	18	19	20	21	22
Differentiation	0	0	0	0	0	0	0	0	0	0	0	1	1	1	1	1	1	1	1	1	1	1
	0	1	0	0	0	0	0	0	0	0	0	1	1	1	1	1	1	1	1	1	1	1
	1	1	1	0	0	0	0	0	0	0	0	0	1	1	1	1	1	1	1	1	1	1
	1	1	1	1	0	0	0	0	0	0	0	0	1	1	1	1	1	0	1	1	1	1
	1	1	1	1	0	1	0	0	0	0	0	0	1	1	1	0	1	0	1	1	1	1
	1	1	1	1	0	1	1	0	0	0	1	0	1	1	1	0	0	0	1	1	1	1
	1	1	1	1	0	1	1	0	0	0	1	0	1	1	1	0	0	0	0	1	1	1
	1	1	1	1	1	1	1	0	1	0	1	0	1	1	1	0	0	0	0	1	1	0
	1	1	1	1	1	1	1	1	1	1	1	0	1	1	1	0	0	0	0	1	1	0
	1	1	1	1	1	1	1	1	1	1	1	0	1	0	1	0	0	0	0	1	1	0
	1	1	1	1	1	1	1	1	1	1	1	0	1	0	1	0	0	0	0	0	1	0
Stem cell	1	1	1	1	1	1	1	1	1	1	1	0	0	0	0	0	0	0	0	0	0	0
