# Supplementary material for: Quantifying Cell Fate Decisions for Differentiation and Reprogramming of a Human Stem Cell Network: Landscape and Biological Paths
Source: PLoS Comput Biol. 2013 Aug 1;9(8):e1003165. doi: 10.1371/journal.pcbi.1003165 (PMC3731225; doi:10.1371/journal.pcbi.1003165)
Supplement: Table S6 — Differentiation path as activation strength changed characterized by high/low expression level of 22 marker genes in Figure 5 of main text. The line of gene ID represent the corresponding genes in Table S1. From the line differentiation to line stem cell, every line represents a cellular states. Stem cell represent the stem cell states, and differentiation represents differentiation states. Intermediate represents the intermediate double low state (both stem cell marker and differentiation marker genes have low expression level). 1 denotes high expression level, and 0 represents low expression level. (PDF) [file pcbi.1003165.s010.pdf]

Table.S 6. Differentiation path as activation strength a changed characterized by high/low expression level of 22 marker genes in Figure 5 of main text. The line of gene ID represent the corresponding genes in Table S1. From the line differentiation to line stem cell, every line represents a cellular states. Stem cell represent the stem cell states, and differentiation represents differentiation states. Intermediate represents the intermediate double low state (both stem cell marker and differentiation marker genes have low expression level). 1 denotes high expression level, and 0 represents low expression level.

gene ID	1	2	3	4	5	6	7	8	9	10	11	12	13	14	15	16	17	18	19	20	21	22
Stem cell	1	1	1	1	1	1	1	1	0	1	1	0	1	0	0	0	0	0	0	0	0	0
	1	1	1	1	1	1	0	1	1	1	1	0	0	0	0	0	0	0	0	0	0	0
	1	1	1	1	1	1	0	1	1	1	1	0	0	1	0	0	0	0	1	0	0	0
	1	1	1	1	1	1	0	1	1	1	1	1	0	0	0	0	0	0	1	0	0	0
	1	1	1	1	1	1	1	1	0	1	1	1	0	0	0	0	0	0	1	0	0	0
	1	1	1	1	1	1	1	1	1	1	1	0	0	0	0	0	0	0	0	0	0	0
	1	1	1	1	1	1	1	1	1	1	1	1	0	0	0	0	0	0	0	0	0	0
	1	1	1	1	1	1	1	1	1	1	1	0	1	0	0	0	0	0	0	0	0	0
	1	0	1	1	1	1	1	1	1	1	1	0	0	0	0	0	0	0	0	0	0	0
	1	1	1	1	1	1	1	1	0	1	1	0	0	0	0	0	0	0	1	0	0	0
	1	0	1	0	1	1	1	1	1	0	1	0	0	0	0	0	0	0	0	0	0	0
	1	0	1	1	1	1	1	1	0	0	1	0	1	0	0	0	0	0	0	0	0	0
	0	0	0	0	0	1	0	1	1	1	1	1	1	0	0	0	0	0	0	0	0	1
	0	0	1	1	0	0	0	1	1	0	0	1	0	0	0	0	0	0	0	0	0	0
	1	0	1	1	0	0	0	1	1	1	0	0	0	0	0	0	0	0	1	0	1	0
	0	1	1	1	0	1	0	1	1	1	1	0	0	0	0	0	0	0	0	0	0	0
	0	0	1	1	0	1	0	1	0	1	0	0	0	0	0	0	0	0	0	0	1	0
	1	0	1	1	0	1	0	1	0	1	1	1	0	1	0	0	0	1	0	0	1	0
	1	0	1	1	0	1	0	1	0	1	0	1	0	1	0	0	0	0	0	0	0	0
	0	1	1	1	0	0	0	1	1	1	1	1	0	1	0	0	0	0	0	0	0	0
	1	0	1	1	1	0	1	1	1	1	1	0	0	0	0	0	0	0	0	0	0	0
	1	1	1	1	1	1	0	1	1	1	1	0	0	0	0	0	0	0	0	0	0	0
	1	0	1	1	0	1	0	1	1	1	1	0	0	0	0	0	0	0	1	0	0	0
	1	1	1	1	1	1	1	1	1	1	1	1	0	0	0	0	0	0	0	0	1	0
	1	0	1	1	1	1	0	1	1	1	1	0	0	0	0	0	0	0	0	0	0	0
	1	1	1	1	1	1	0	1	1	1	1	0	1	0	0	0	0	0	0	0	0	0
	1	1	1	1	1	1	1	1	1	1	1	1	0	0	0	0	0	0	0	0	0	0
	1	1	1	1	1	1	0	1	1	1	1	1	0	0	0	0	0	0	0	0	0	0
	1	1	1	1	1	1	1	1	1	1	1	1	1	0	0	0	0	0	0	0	0	0
	1	1	0	0	1	1	1	1	1	1	1	1	0	0	0	0	0	0	0	0	0	0
	1	1	0	0	1	1	1	1	0	1	1	0	0	0	0	0	0	0	0	0	0	0
	1	0	1	0	1	1	1	1	0	1	1	1	0	0	0	0	0	0	0	0	0	0
	1	1	1	0	1	1	1	1	0	1	0	1	0	0	1	0	0	0	0	0	0	0
	1	0	1	0	1	1	1	1	0	1	1	1	0	0	1	0	0	0	0	0	0	0
	1	1	1	0	1	1	1	1	1	1	0	0	0	0	1	0	0	0	0	0	0	0
	1	1	1	1	1	1	1	1	0	1	1	0	0	0	0	0	0	0	0	0	0	0
	1	1	1	0	1	1	1	1	1	1	1	1	1	0	0	0	0	0	0	0	0	0
	1	1	1	1	1	1	1	1	0	1	1	0	0	0	0	0	0	0	0	0	0	0
	1	1	1	1	1	1	1	1	0	1	1	0	1	0	0	0	0	0	0	0	0	0

Continued. . .

gene ID	1	2	3	4	5	6	7	8	9	10	11	12	13	14	15	16	17	18	19	20	21	22
	1	1	1	0	1	1	1	1	1	1	1	1	1	0	0	0	0	0	0	0	0	0
	1	1	1	1	1	1	1	1	1	1	1	0	0	0	0	0	0	0	1	0	0	0
	1	1	1	1	1	1	0	1	1	1	1	0	0	0	0	0	0	0	0	0	0	0
	1	1	1	1	1	1	0	1	0	1	0	0	0	1	0	0	0	0	0	0	0	0
	1	0	0	1	1	1	0	1	0	1	1	0	0	1	0	0	0	0	0	0	0	0
	1	0	0	1	1	1	0	1	1	0	1	0	1	0	0	0	0	0	0	0	0	0
	1	0	0	1	0	1	0	1	0	1	1	0	0	1	0	0	0	0	0	0	0	0
	1	1	1	0	1	1	0	1	1	0	1	1	0	1	0	0	0	0	0	0	0	0
	1	0	1	0	1	1	0	1	1	1	1	1	0	0	0	0	0	0	0	0	0	0
	1	0	1	1	1	1	0	1	0	1	1	1	0	1	0	0	0	0	1	0	0	0
	1	0	1	0	1	1	0	1	1	1	1	0	1	1	0	0	0	0	0	0	0	0
Intermediate	0	0	0	0	0	1	0	1	1	1	1	0	1	1	0	0	0	0	0	0	0	1
Intermediate	0	0	0	0	0	0	0	1	1	0	0	1	0	1	1	0	0	0	0	0	1	1
	0	0	0	0	0	0	0	1	0	0	0	1	0	1	1	0	0	1	0	0	0	1
	0	0	0	0	0	0	0	1	1	0	0	1	0	1	1	0	0	1	0	0	1	1
	0	0	0	0	0	0	0	1	0	0	0	1	0	1	1	0	1	0	1	1	1	1
	0	0	0	0	0	0	0	0	0	0	0	1	0	1	1	1	1	1	1	1	1	1
	0	0	0	0	0	0	0	0	0	0	0	1	1	1	1	1	1	1	1	1	1	1
	0	0	0	0	0	0	0	0	0	0	0	1	1	1	1	1	1	1	1	1	1	1
	0	0	0	0	0	0	0	1	0	0	0	1	1	1	1	1	1	1	0	1	1	1
	0	0	0	0	0	0	0	0	0	0	0	1	0	1	1	1	1	1	1	1	1	1
	0	0	0	0	0	0	0	0	0	0	0	1	1	1	1	1	1	1	1	1	1	1
	0	0	0	0	0	0	0	0	0	0	0	1	1	1	1	1	1	1	1	1	1	1
	0	0	0	0	0	0	0	0	1	0	0	1	1	1	1	1	1	1	1	1	1	1
	0	0	0	0	0	0	0	0	0	0	0	1	1	1	1	1	1	1	1	1	1	1
	0	0	0	0	0	0	0	0	1	0	0	1	1	1	1	1	1	1	1	1	1	1
	0	0	0	0	0	0	0	0	0	0	0	1	1	1	1	1	1	1	1	1	1	1
	0	0	0	0	0	0	0	0	0	0	1	1	1	1	1	1	1	1	1	1	1	1
	0	1	0	0	0	0	0	0	0	0	0	1	1	1	1	1	1	1	1	1	1	1
	0	1	0	1	0	0	0	0	0	0	0	1	0	1	1	1	1	1	1	1	1	1
	0	1	0	1	0	0	0	0	0	0	0	1	1	1	1	1	1	1	1	1	0	1
	0	0	0	0	0	0	0	0	1	0	0	0	1	1	1	1	1	1	1	1	1	1
	0	0	0	0	0	0	0	0	0	0	0	1	1	1	1	1	1	1	1	1	1	1
	0	0	0	0	0	0	0	0	0	0	0	1	1	1	1	1	1	1	1	1	1	1
	0	0	0	0	0	0	0	0	0	0	0	1	1	1	1	1	1	1	1	1	1	1
	0	0	0	0	0	0	0	0	0	0	0	1	0	1	1	1	1	1	1	1	1	1
	0	0	0	0	0	0	0	0	0	0	0	1	0	1	1	1	1	1	1	1	1	1
	0	0	0	0	0	0	0	0	0	0	0	1	0	1	1	1	1	1	1	1	1	1
	0	0	0	0	0	0	1	0	1	0	1	1	0	1	1	1	1	1	1	1	1	1
	0	0	0	0	0	0	1	0	1	0	1	1	0	1	1	1	1	1	1	1	1	1
	0	0	0	0	0	0	1	0	0	0	0	1	0	1	1	1	1	1	1	1	1	1
	0	0	0	0	0	0	0	0	0	0	0	1	1	1	1	1	1	1	1	1	1	1
	0	0	0	0	0	0	0	0	0	0	1	1	1	1	1	1	1	1	0	1	1	1
	0	0	0	0	0	0	0	0	1	0	0	1	1	1	1	1	1	1	1	1	1	1
	0	0	0	0	0	0	0	0	0	0	0	0	1	1	1	1	1	1	0	1	1	1
	0	0	0	0	0	0	0	0	0	0	0	0	1	1	1	1	1	1	1	1	1	1
	0	0	0	0	0	0	0	0	0	0	1	1	1	1	1	1	1	1	1	1	1	1

Continued...

gene ID	1	2	3	4	5	6	7	8	9	10	11	12	13	14	15	16	17	18	19	20	21	22
	0	0	0	0	0	0	0	0	0	0	0	1	0	1	1	1	1	1	1	1	0	1
	0	0	0	0	0	0	0	0	0	0	0	0	0	1	1	1	1	1	1	1	1	1
	0	0	0	0	0	0	0	0	1	0	0	0	0	1	1	1	1	1	1	0	1	1
	0	0	0	0	0	0	0	0	0	0	0	0	0	1	1	1	1	1	1	1	1	1
	0	0	0	0	0	0	0	0	0	0	1	0	0	1	1	1	1	1	1	1	1	1
	0	0	0	0	0	0	0	0	0	0	1	1	1	1	1	1	1	1	1	1	1	1
	0	0	0	0	0	0	0	0	0	0	1	1	1	1	1	1	1	1	1	1	1	1
	0	0	0	0	0	0	0	0	1	0	0	1	1	1	1	1	1	1	1	1	1	1
	0	1	0	0	0	0	0	0	1	0	0	1	1	1	1	1	1	0	0	1	1	1
	0	0	0	0	0	0	1	0	0	0	0	1	1	1	1	1	1	1	1	1	1	1
	0	0	0	0	0	0	0	0	0	0	1	1	0	1	1	1	1	1	1	1	1	1
	0	0	0	0	0	0	0	0	0	0	0	1	0	1	1	1	1	1	1	1	1	1
	0	0	0	0	0	0	0	0	0	0	0	1	1	1	1	1	1	1	1	1	1	1
Differentiation	0	0	0	0	0	0	1	0	1	0	1	1	1	1	1	1	1	1	1	1	1	1
