# Supplementary material for: Quantifying Cell Fate Decisions for Differentiation and Reprogramming of a Human Stem Cell Network: Landscape and Biological Paths
Source: PLoS Comput Biol. 2013 Aug 1;9(8):e1003165. doi: 10.1371/journal.pcbi.1003165 (PMC3731225; doi:10.1371/journal.pcbi.1003165)
Supplement: Text S1 — Supplementary results and methods. (PDF) [file pcbi.1003165.s012.pdf]

Quantifying Cell Fate Decisions for Differentiation and Reprogramming of a Human Stem Cell Network: Landscape and Biological Paths

Chunhe Li[1], Jin Wang[1,2,*]
1 Department of Chemistry and Physics, State University of New York at Stony Brook, Stony Brook, NY, USA
2 State Key Laboratory of Electroanalytical Chemistry, Changchun Institute of Applied Chemistry, Chinese Academy of Sciences, Changchun, Jilin, China
∗ E-mail: jin.wang.1@stonybrook.edu

Supplementary Results

Figure S1 gives a flowchart for the methods that we used to acquire landscape, probabilistic flux, barrier heights, MFPT (mean first passage time) dynamic paths and the sensitivity analysis.

In order to exhibit the landscape of the complete 52 dimensional network, we also used Langevin dynamics method to obtain landscape (Figure S2). For a 52 dimensional system, for visualization, we harnessed $RMSD$ (root mean squared distance) as the coordinate to reduce the dimensionality to 2 dimension ($RMSD = \sqrt{\sum_i^N (x_i - x_i^{ref})^2}$, N is the number of variables, and x_i^{ref} is the reference state, here we chose two potential minima as the reference states). $RMSD$ represents the distance between a state point and reference point in state space. In this way, from 52-dimensional trajectory, we can generate two new coordinates $RMSD1$ and $RMSD2$, separately representing the distance from a state point to the reference state 1 (the potential minimum of stem cell attractor) and the reference state 2 (the potential minimum of differentiation state attractor). We can find that the landscapes using RMSD method based on Langevin dynamics (Figure S2) possess the similar dynamics compared with using NANOG and GATA6 as the coordinates (Figure 2 in main text) based on the self consistent approximation. This shows that the two dimensional projection of landscape in NANOG and GATA6 state space can reflect the major dynamics of the full 52-dimensional gene network.

We found that the landscape is critically influenced by the activation constant a. When a increases the stem cell state will be dominant and when a decline the differentiation state will be dominant. Figure S2 show the landscape of the stem cell network when activation constant a is changed, separately corresponding to $a = 0.5$,$a = 0.38$,$a = 0.36$,$a = 0.3$. Landscape comparisons illustrated that $a = 0.3$ represents the case of prominent differentiation state (right attractor), and when $a = 0.5$ the stem cell state (left attractor) dominates. It also shows that when a gradually decreases from 0.5 to 0.3, the stem cell state becomes less and less stable and the differentiation state becomes more and more stable until being dominant, demonstrating that the system of stem cell experiences a transition from stem cell state to differentiation state with activation strength a decreased.

For sensitivity analysis, we firstly exploited the self consistent approximation method to obtain those top parameters - that is, by finding those parameters affecting barrier heights of the system critically. Specifically, we changed the value of each of the activation and repression constant A_{ji} and B_{ji} (Eq. (2), the parameters A_{ji} and B_{ji} and rp_{ji} are only used for the sensitivity analysis) by giving a perturbation level pl (here, the value of pl is chosen as : -0.5, -0.2, -0.1, 0.1, 0.2, 0.5, and the default parameter value is: $A_{ji} = 0.37, B_{ji} = 0.5$) as the percentage to change. Then for every mutation of parameters we compared the change of the landscape topography in terms of the barrier heights for both differentiation ΔU_{SP} and reprogramming ΔU_{SD}. Figure S3 show barrier change results when pl =0.5 in terms of self consistent approximation method. Figure S3A present the results for 84 activation parameters (change activation constant A_{ji} to 150% of its default value) and Figure S3B present the results for 39 repression parameters (change repression constant B_{ji} to 150% of its default value). Blue stairs represent the barrier for differentiation process U_{SP} ($U_{differentiation}$) for different mutations, red stairs represent the barrier for

differentiation state U_{SD} ($U_{reprogramming}$) for different mutations. X axis in A represent all 84 activation links, and X axis in B represent all 39 repression links. From Figure S3, we can see that most of the parameters only have small influence to the barrier heights compared with the default value. In this way, we acquired 14 top activation links (absolute value of barrier change is larger than 3 in Figure S3A) and 6 top repression links (absolute value of barrier change is larger than 1 in Figure S3B).

In the following, we employed the Langevin dynamics to further obtain the change of barrier heights for the above top 20 parameters, because by the Langevin dynamics the landscape of the system can be acquired directly by the statistics of the trajectories of the system - not through approximation. Figure 6 in main text shows the results of the sensitivity analysis for the 20 top parameters. Figure 6A in main text shows the results for 6 repression links (changing parameter rp_{ji}), and Figure 6(B) in main text shows the results for 14 activation links (changing parameter A_{ji}). In Figure 6, every parameter is perturbed to 200% compared to its default value ($A_{ji} = 0.37, rp_{ji} = 1$). Here, we introduce another parameter rp_{ji}, which is only used in sensitivity analysis section (Figure 6 in main text). The reason is the activation constant A_{ji} and repression constant B_{ji} actually represent the relative weight of activation or repression link (or maximum protein synthesis rate separately for activation item and repression item) in the driving force equation (Eq (1) or Eq (2)). The increase of parameter B_{ji} cannot really reflect the increase of inhibition of gene j to gene i, as we can see from Eq. (2) that the increase of B_{ji} actually will lead to activation of gene i. Therefore, we introduce the parameter rp, whose increase can really represent the increase of inhibition of gene j to gene i. Then we did sensitivity analysis with respect to the activation constant A_{ji} and the new repression constant rp_{ji} in terms of Langevin dynamics (Figure6 in main text).

$$F_i = -k * X_i + \sum_{j=1}^{m1} \frac{a * {X_j}^n}{S^n + {X_j}^n} + \sum_{j=1}^{m2} \frac{b * S^n}{S^n + {X_j}^n} \tag{1}$$

$$F_i = -k * X_i + \sum_{j=1}^{m1} \frac{A_{ji} * {X_j}^n}{S^n + {X_j}^n} + \sum_{j=1}^{m2} \frac{B_{ji} * S^n}{S^n + rp_{ji}{X_j}^n} \tag{2}$$

Self Consistent Mean Field Approximation

The time evolution the dynamical systems are governed by the diffusion equations. Given the system state $P(X_1, X_2, ..., X_n, t)$, where $X_1, X_2, ..., X_n$ is the concentration or populations of molecules or species, we expected to have N-coupled differential equations, which are difficult to solve. Following a self consistent mean field approach [1–3], we split the probability into the products of individual ones: $P(X_1, X_2, ..., X_n, t) \sim \prod_i^n P(X_i, t)$ and solve the probability self-consistently. This effectively reduces the dimensionality from M^N to $M \times N$, and thus makes the problem computationally tractable.

However, for the multi-dimensional system, it is still hard to solve diffusion equations directly. We can start from moment equations and then simply assume specific probability distribution based on physical argument, meaning that we give some specific connections between moments. In principle, once we know all moments, we can construct the probability distribution. For example, Poisson distribution has only one parameter, so we may calculate all other moments from the first moment, that is the mean. Here we use gaussian distribution as approximation, then we need two moments, mean and variance.

Let us begin from one dimensional diffusion equation [4, 5]:

$$\frac{\partial P(x,t)}{\partial t} = -\frac{\partial}{\partial x}[F(x)P(x,t)] + D\frac{\partial^2}{\partial x^2}[d(x)P(x,t)] \tag{3}$$

Here $F(x)$,$d(x)$ is "drift and diffusion part". For this equation, in weak noise $D \ll 1$, we divide x to two part:

$$\begin{aligned} x &= x(t) + \sqrt{D}y \\ x(t) &= \langle x \rangle = \int xP(x,t)dt \\ y &= o(1) \end{aligned} \tag{4}$$

Then, we expand Eq. (3) for D, and discuss all moment equations of the system. First, multiplying x in two sides of Eq. (3), and integrating x in whole space, we can get the first moment equation of x.

$$\dot{x}(t) = \langle F(x) \rangle \tag{5}$$

Bring equation (4) to equation (5), we can get:

$$\dot{x}dt = F[x(t)] + \frac{D}{2}\frac{\partial^2 F[x(t)]}{\partial x(t)^2}\sigma(t) + o(D^{\frac{3}{2}}), \sigma(t) = \langle y^2 \rangle. \tag{6}$$

Using x^2 to multiply Eq. (3) in two sides, in the same way we can get:

$$2x(t)\dot{x}(t) + D\dot{\sigma}(t) = 2x(t)(F[x(t)] + D\frac{\partial^2 F[x(t)]}{\partial x(t)^2}\sigma(t)) + 2D\frac{\partial F[x(t)]}{\partial x(t)}\sigma(t) + 2Dd[x(t)] + o(D^{\frac{3}{2}}) \tag{7}$$

Bring Eq. (6) to Eq. (7), we can finally get:

$$\dot{\sigma}(t) = 2\frac{\partial F[x(t)]}{\partial x(t)}\sigma(t) + 2d[x(t)] + o(\sqrt{D}). \tag{8}$$

When diffusion coefficient D is small, if $D\sigma(t)$ is not big enough to 1/D level, the Eq. (6),(8) could be approximated to:

$$\dot{x}(t) = F[x(t)] \tag{9}$$

$$\dot{\sigma}(t) = 2\frac{\partial F[x(t)]}{\partial x(t)}\sigma(t) + 2d[x(t)]. \tag{10}$$

For multi-dimensional case, the moment equations can be approximated to [4, 5]:

$$\dot{\bar{\mathbf{x}}}(\mathbf{t}) = \mathbf{F}[\bar{\mathbf{x}}(\mathbf{t})] \tag{11}$$

$$\dot{\sigma}(t) = \sigma(t)\mathbf{A^T}(\mathbf{t}) + \mathbf{A}(\mathbf{t})\sigma(\mathbf{t}) + \mathbf{2D}[\bar{\mathbf{x}}(\mathbf{t})]. \tag{12}$$

Here,$\mathbf{x}, \sigma(t)$, and $\mathbf{A}(t)$ are vectors and tensors, and $\mathbf{A^T}(t)$ is the transpose of $\mathbf{A}(t)$. The matrix elements of A is $A_{ij} = \frac{\partial F_i[X(t)]}{\partial x_j(t)}$. In terms of this equation, we can solve $\mathbf{x}(t)$ and $\sigma(t)$. Here, we consider only diagonal elements of $\sigma(t)$ from mean field splitting approximation. Therefore, the evolution of probabilistic distribution for each variable could be acquired using the mean and variance based on gaussian approximation:

$$P(x,t) = \frac{1}{\sqrt{2\pi\sigma(t)}} \exp -\frac{[x - \bar{x}(t)]^2}{2\sigma(t)} \tag{13}$$

The probability obtained above corresponds to one fixed point or basin of attraction. If the system allows multistability, then there are several probability distributions localized at every basin of attraction, but with different variations. Therefore, the total probability is the weighted sum of all these probability distributions. The weighting factors (w_1, w_2) are the size of the basin, representing the relative size of different basin of attraction. For example, for a bistable system, the probability distribution takes the form: $P(x,t) = w_1 P^a(x) + w_2 P^b(x)$, here $w_1 + w_2 = 1$.

Finally, once we have the total probability, we can construct the potential landscape by the relationship with the steady state probability: $U(x) = -lnP_{ss}(x)$. In the gene regulatory network system, every parameter or link contributes to the structure and dynamics of the system, which is encoded in the total probability distribution, or the underlying potential landscape.

For nonequilibrium gene regulatory systems, the driving force F can not be written as the gradient of potential U, like the equilibrium case. In general, F can be decomposed into a gradient of the potential and a curl flux force linking the steady state flux $\mathbf{J_{ss}}$ and the steady state probability P_{ss} [2,6]($\mathbf{F} = +\mathbf{D}/P_{ss} \cdot \frac{\partial}{\partial \mathbf{x}} P_{ss} + \mathbf{J_{ss}}(\mathbf{x})/P_{ss} = -D\frac{\partial}{\partial \mathbf{x}} U + \mathbf{J_{ss}}(\mathbf{x})/P_{ss}$). P_{ss} denotes steady state probability and potential U is defined as $U = -lnP_{ss}$. The probability flux vector $\mathbf{J}$ of the system in concentration or gene expression level space $\mathbf{x}$ is defined as [5]: $\mathbf{J}(\mathbf{x},t) = \mathbf{F}P - \mathbf{D} \cdot \frac{\partial}{\partial \mathbf{x}} P$.

In the 52-dimensional protein concentration space, it's hard to visualize 52-dimensional probabilistic flux. Approximately, we explored the associated 2-dimensional projection of flux vector: $J_1(x_1, x_2, t) = F_1(x_1, x_2)P - D\frac{\partial}{\partial x_1} P$ and $J_2(x_1, x_2, t) = F_2(x_1, x_2)P - D\frac{\partial}{\partial x_2} P$.

Given a GRN with m nodes and their mutual regulation directions (activation or repression), according to Eq (1) in main text, we can write down the m ODE separately representing the driving force F_i for the ith gene. Then, according to Eq (3) and Eq(4) in main text (here Eq (3) has the same form as Eq (1) as the driving force F), for a m variable system, we have m+m=2m ODEs, in which m ODEs represent equations for mean value (first moment) and the other m ODEs represent equations for variance (second moment). For our system we have 104 ODEs, we used Mathematic 7.0 software to solve these 104 ODEs to obtain the solutions for the first moment and second moment (and). When solving ODEs, by giving a large number (100000) of different random initial values for 52 variables, we can obtain different stable solutions for different initial value, i.e. we can acquire multi-stable solution (bistable in this work). For the Gaussian approximation, we determine the weights w_i by giving a large number of random initial conditions for ODEs to find solution, and then collect the statistics for different solution. For example, for a bistable system, if 10% initial condition goes to the first steady state, and 90% initial condition goes to the second steady state, then the weight w_1 for the first basin is 0.1 and w_2 for the second basin is 0.9. The multistability comes from the solution of 52 ODEs giving a large number (100000) of random initial values. We give large number of random different initial conditions for ODEs for solution at a fixed parameter set. By collecting the statistics of the solution, we can determine if the system is monostable or bistable or mutistable at current parameter region.

To validate the Gaussian approximation method, we provided the landscape results from Gaussian distribution approximation of the 2-dimensional case for GATA1/PU1 [7,8], and made comparisons for this 2-dimension case between Gaussian approximation method and Langevin dynamics method (Figure S4). Figure S4 show the landscape comparisons for 3 different parameter values (a=1.2 for first column, a=1 for second column, a=0.2 for the third column). The first row is from Gaussian approximation and the second row is from Langevin dynamics. We can see that the landscapes from Gaussian approximation preserve the similar global properties (the number of attractors, the relative stability of basin of attractions) as the Langevin dynamics method.

The motivation of our self-consistent method of splitting the variables is to reduce the dimensionality of the large networks from exponential number of degrees of freedom to polynomial number of degrees of freedom (from M^N to $M*N$, here M is the number of how many different value every protein concentration variable can have and N is the number of protein species). Our method is not simply splitting the variables as an independent product but with a mean field type of approximation. In other words, even though

the form of the probability is a product like $P(x_1, ..., x_q, ..., x_N) = P(x_1)...P(x_q)...P(x_N)$, each of the component $P(x_q)$ is not entirely independent with the others. The effect of the interactions of other components is taken into account by the mean field or average of others on this particular component. In order to solve each individual $P(x_q)$, a self consistent equation for $P(x_q)$ has to be solved taking into account of the mean field effects from averaging the other components. In other words, the interactions among different components is taken into account approximately by the self consistent way of solving the each component $P(x_q)$ in the back ground of the average effects of others. The self consistent method has been applied to multi-electron atom and multi atom molecule studies [9]. The results are usually in reasonable agreements with experiments.

In our current work, for 52 dimensional system, we have 52 Gaussians each for a variable (52 dimensional probability distribution). To exhibit the results in 2-dimensional space, we integrated out the other 50 variables and left two variables NANOG and GATA6.

Paths for Differentiation and Reprogramming from Discretized Dynamics

The landscape in Figure 2 of main text only is the 2-dimensional projection of the whole 52 dimensional state space. In order to demonstrate the cell states and the transitions between different cell types in the complete state space, we projected the expression level of the 52 gene variables to binary states, and acquired discretized dynamics results of the network (Figure 3 in main text).

We first used the Langevin dynamics to obtain the stochastic dimensionless trajectories of the 52 dimensional system. Then the trajectory is converted to discrete trajectories by setting the value ((maximum value - minimum value)/2 + minimum value) of every variable as the cutoff (cutoff is chosen so that two up/down states are well separated), i.e. the value higher than the cutoff is set to 1 (indicating high expression), while the value lower than the cutoff is set to 0 (indicating low expression). So, we can obtain the discrete trajectories for 52 variables of the system. For a 52 dimension system, there will be 2^{52} states even in discrete case (every variable has two value, 1 represent high expression, 0 represent low expression), which cannot be handled computationally. So, we chose the major 22 marker genes to present the discrete system, which has $2^{22} = 4194304$ states. For example, the stem cell state is represented by the binary number 1111111111100000000000 (representing expression level from gene 1 to gene 22, 1 for high expression, 0 for low expression), and for the differentiation state, it is represented by 0000000000011111111111. By the statistics for the discrete trajectory, we can obtain the appearing probability separately for 2^{22} different states. To present the results, we set a probability cutoff 0.0002 (only states with higher probability than 0.0002 are chosen, the cutoff is chosen so that the major states can be presented in a figure, not too many or too few states, i.e. we only demonstrate the states and paths with higher probability). Figure 3 in main text shows the differentiation and reprogramming process represented by 313 cell states (nodes) and 329 transition paths (edges) between the different cell states. We believe that these 313 states with higher probability can capture the major states and regulation dynamics of the system. The sizes of nodes and edges are separately proportional to the occurrence probability of the corresponding states and paths. Red nodes represent states which are closer to stem cell states, and blue nodes represent states which are closer to differentiation states. Especially, we acquired the dominant kinetic paths as the biological paths from path integral formulism , which are shown as green and magenta paths (Figure 3 in main text) separately for differentiation and reprogramming process (see Table S4 and Table S5 for detailed paths).

From Table S4, monitoring the differentiation process according to certain vital marker genes NANOG (column 3), GATA6 (column 16) and CDX2 (column 22), we can see that the differentiation process experiences a transition from the stem cell state (high NANOG/low GATA6/low CDX2) to a intermediate state (IM1, low NANOG/low GATA6/low CDX2), and then to another intermediate state (low

NANOG/low GATA6/high CDX2), and eventually to the differentiation state (low NANOG/high GATA6/high CDX2). This indicates the importance of NANOG to the maintenance of pluripotency. For differentiation proceeding, the cell needs to firstly impair the expression of NANOG, further downregulate other stem cell marker genes which are promoted by NANOG, and finally reach the differentiation state (GATA6 dominant). For the reprogramming path in Table S5, we can see that the cell experiences a transition from the differentiation state (low NANOG/high GATA6/high CDX2), to an intermediate state (IM2, high NANOG/high GATA6/high CDX2), to another intermediate state (high NANOG/low GATA6/high CDX2), and finally to the stem cell state (high NANOG/low GATA6/low CDX2). This might imply that in the reprogramming process the cell first opens the key stem cell marker genes NANOG by the change of regulation strength between key maker genes, then other stem cell marker genes gradually acquire high expression level due to the activation regulation of NANOG to them. Finally the cell reach the stem cell state, because the stem cell marker genes which have been activated repress strongly the differentiation marker genes (such as GATA6 and CDX2). The biological paths can be validated by related experiments, and we expect that it can be used to guide the design of new strategies for cellular differentiation and reprogramming.

As we did for the dominant path, we also monitored the differentiation and reprogramming kinetic paths with the activation strength a changed (separately shown in Table S6 and Table S7) in terms of certain key marker genes NANOG, GATA6, and CDX2. Similar to the analysis about dominant paths from path integrals, we can find that for the differentiation process the cell experiences an intermediate state (low NANOG/low GATA6/low CDX2 or low stem cell marker/low differentiation marker) along the path from the stem cell state to the differentiation state. For the reprogramming path, we can see that the cell also experiences an intermediate state (high NANOG/high GATA6/high CDX2, or high stem cell marker/high differentiation marker) along the path from the differentiation state to the stem cell state. These results have the consistent predictions with the dominant path analysis, which is that the cellular differentiation needs to experience an intermediate double low state (both stem cell marker genes and differentiation marker genes have low expression level), and the cellular reprogramming needs to experience an intermediate double high state (both stem cell marker genes and differentiation marker genes have high expression level). We expect that these predictions can be tested by experiments in the future, as well as help to design the differentiation and reprogramming strategies.

Path Integrals

In the cell, there exist external noise and intrinsic noise, which can be significant to the dynamics of the system [10, 11]. Therefore, a network of chemical reactions in noisy fluctuating environments can be addressed by:$\dot{\mathbf{x}} = \mathbf{F}(\mathbf{x}) + \zeta$. Here, $\mathbf{x} = (x_1(t), x_2(t), ..., x_{52}(t))$ represents the vector of protein concentration. $\mathbf{F}(\mathbf{x})$ is the vector for the driving force of chemical reaction. ζ is Gaussian noise term whose autocorrelation function is $< \zeta_i(\mathbf{x}, t)\zeta_j(\mathbf{x}, 0) >= 2D\delta(t)$, and D is diffusion coefficient matrix.

The dynamics for the probability of starting from initial configuration $\mathbf{x}_{initial}$ at t=0 and ending at the final configuration $\mathbf{x}_{final}$ at time t, in terms of the Onsager-Machlup functional, can be formulated [8,12,13] as: $P(\mathbf{x}_{final}, t, \mathbf{x}_{initial}, 0) = \int \mathbf{D}\mathbf{x} \exp[-\int dt(\frac{1}{2}\nabla\cdot\mathbf{F}(\mathbf{x})+\frac{1}{4}(d\mathbf{x}/dt-\mathbf{F}(\mathbf{x}))\cdot\frac{1}{\mathbf{D}(\mathbf{x})}\cdot(d\mathbf{x}/dt-\mathbf{F}(\mathbf{x})))] = \int \mathbf{D}\mathbf{x} \exp[-S(\mathbf{x})] = \int \mathbf{D}\mathbf{x} \exp[-\int L(\mathbf{x}(t))dt]$.

$\mathbf{D}(\mathbf{x})$ is the diffusion coefficient matrix. The integral over $\mathbf{Dx}$ denotes the sum over all possible paths from the state $\mathbf{x_{initial}}$ at time $t = 0$ to $\mathbf{x_{final}}$ at time t. The exponent factor gives the weight of each path. Therefore, the probability of network dynamics from initial state $\mathbf{x_{initial}}$ to the final state $\mathbf{x_{final}}$ is equal to the sum of all possible paths with different weights. The $S(\mathbf{x})$ is the action and $L(\mathbf{x}(\mathbf{t}))$ is the Lagrangian or the weight for each path.

The path integrals can be approximated with a set of dominant paths, since each path is exponentially weighted, and the other subleading path contributions are often small and can be neglected. Therefore, the dominant path with the optimal weights can be acquired through minimization of the action or

Lagrangian. In our case, we identify the optimal paths as the biological paths or differentiation and reprogramming paths.

Hamilton-Jacobian (HJ) Framework for Path Integral.

From our path integral formalism, we can evaluate the weights of the kinetic paths. The most probable trajectory can be acquired when the action $S(x)$ is minimized directly. The Lagrangian is written as:

$$L(\mathbf{x}) = \frac{1}{4D}\dot{\mathbf{x}}^2 + V(\mathbf{x}) - \frac{1}{2D}\mathbf{F}(\mathbf{x}) \cdot \dot{\mathbf{x}} \tag{14}$$

and thus the generalized momentum can be written out as: $\mathbf{P}(\mathbf{x}) = \frac{\partial L}{\partial \dot{x}} = \frac{1}{2D}(\dot{\mathbf{x}} - \mathbf{F}(\mathbf{x}))$. In the kinetic system, the Hamiltonian of the system has the form:

$$H(\mathbf{x}) = -L(\mathbf{x}) + \mathbf{P}(\mathbf{x}) \cdot \dot{\mathbf{x}} = E_{eff} \tag{15}$$

According to the above equation, we can obtain $\frac{1}{4D}\dot{\mathbf{x}}^2 - V(\mathbf{x}) = E_{eff}$ and $|\dot{\mathbf{x}}| = \sqrt{4D(E_{eff} + V(\mathbf{x}))}$. After substituting Eq. S2 into the action, we can obtain $S(\mathbf{x}) = \int(\mathbf{P}(\mathbf{x}) \cdot \dot{\mathbf{x}} - H(\mathbf{x}))dt$. We can see that the action characterizing the weights of the paths depends on the values of the Hamiltonian. Specific values of the Hamiltonian correspond to specific values of the final time T. For a fixed Hamiltonian, a corresponding optimal path exits when minimizing the action $S(\mathbf{x})$.

From the least action principle, if the Hamiltonian of the system is constant, the variation of the action, for given initial and final coordinates and initial and final time, is zero. Allowing a variation of the final time T and leaving the initial and the final coordinates fixed, we have $\delta S = -H\delta t$. For a constant Hamiltonian, $\delta S = -E\delta t$. We define $S_0 = \int \mathbf{P}(\mathbf{x}) \cdot \dot{\mathbf{x}}dt$, since $S(\mathbf{x}) = \int(\mathbf{P}(\mathbf{x}) \cdot \dot{\mathbf{x}} - H(\mathbf{x}))$. We find $\delta S_0 = 0$. Thus, the action S_0 is minimized with respect to all the paths satisfying the constant Hamiltonian and passing through the final point at any instant.

For multidimensional questions, the action depends not only the initial and final coordinates but also on the initial and final time. In the HJ framework, we can transform the formulations into a different representation in x space: $S_0 = S_{HJ}(\mathbf{x}) = \int \sum_i \frac{1}{2D}(\dot{\mathbf{x}}_\mathbf{i} - \mathbf{F}_\mathbf{i})dx_i = \int \sum_i p_i(\mathbf{x})dx_i$. Here p_i is the associated momentum. Now the action only depends on the initial and final coordinates. This action can be further simplified and is equivalent to a line integral along a particular one dimensional path l so that $S_{HJ}(\mathbf{x}) = \int \sum_i p_i(\mathbf{x})dx_i = \int p_l dl$ where $p_l = \sqrt{(E_{eff} + V(\mathbf{x}))/D} - \frac{1}{2D}F_l$. This switch from the time-dependent to the Hamiltonian-dependent HJ description [8, 13, 14]. The dominant path connection given initial and final states is obtained by minimizing the action in the HJ representation $S_{HJ} = \int_{x_i}^{x_f}(\sqrt{(E_{eff} + V(\mathbf{x}))/D} - \frac{1}{2D}F_l)dl$, where dl is an infinitesimal displacement along the path trajectory. E_{eff} is a free parameter that determines the total time elapsed during the transition.

In the current work, for simplification we chose $E_{eff} = -V_{min}(x)$, which is the effective potential by minimizing $V(\mathbf{x})$, and corresponding to the longest kinetic time. Finally, the optimal paths were obtained by minimizing the discrete target function:

$$S_{HJ} = \sum_{n}^{N-1}(\sqrt{(E_{eff} + V(n))/D} - \frac{1}{2D}F_l(n))\Delta l_{n,n+1} + \lambda P \tag{16}$$

where

$$\begin{aligned} P &= \sum_{i}^{N-1}(\Delta l_{i,i+1} - <\Delta l>)^2 \\ (\Delta l)^2_{n,n+1} &= \sum_i (\mathbf{x}_i(n+1) - \mathbf{x}_i(n))^2 \\ F_l(n) &= \sum_i \mathbf{F_i}(\mathbf{x}(n))(\mathbf{x_i}(n+1) - \mathbf{x}_i(n))/\Delta l_{n,n+1} \\ V(n) &= \sum_i (\frac{1}{4D}\mathbf{F}^2(\mathbf{x_i}) + \frac{1}{2}\sum_j \frac{\partial \mathbf{F_j}(\mathbf{x_i})}{\partial \mathbf{x_j}}) \end{aligned} \quad (17)$$

Here, $\Delta l_{n,n+1}$ is the Euclidean measure of the nth elementary path step, and P is a penalty function, which keeps all the length elements close to their average and becomes irrelevant in the continuum limit. The minimization of the discrete HJ effective action was performed by applying a simulated annealing algorithm or the conjugate gradient algorithm. In this study, we chose the discrete steps n as 20, and the diffusion coefficient is chosen as 0.01. In Figure 2 and Figure 6 of main text, we projected the 52-dimensional path to 2-dimensional state space with respect to stem cell marker gene NANOG and differentiation marker gene GATA6 as the biological paths. In Table S4 and Table S5, we showed the 22-dimensional discrete paths for differentiation and reprogramming characterized by 22 marker genes.

References

1. Sasai M, Wolynes P (2003) Stochastic gene expression as a many-body problem. Proc Natl Acad Sci U S A 100: 2374-2379.

2. Wang J, Li CH, Wang EK (2010) Potential and flux landscapes quantify the stability and robustness of budding yeast cell cycle network. Proc Natl Acad Sci USA 107: 8195-8200.

3. Li CH, Wang J, Wang EK (2011) Potential landscape and probabilistic flux of a predator prey network. PLoS ONE 6: e17888.

4. Hu G (1994) Stochastic Forces and Nonlinear Systems. Shanghai: Shanghai Scientific and Technological Education Press, 68-74 pp.

5. Van Kampen NG (1992) Stochastic processes in Chemistry and Physics. Amsterdam: North Holland, 1 edition, 120-127 pp.

6. Wang J, Xu L, Wang EK (2008) Potential landscape and flux framework of non-equilibrium networks: Robustness, dissipation and coherence of biochemical oscillations. Proc Natl Acad Sci USA 105: 12271-12276.

7. Wang J, Xu L, Wang EK, Huang S (2010) The potential landscape of genetic circuits imposes the arrow of time in stem cell differentiation. Biophys J 99: 29-39.

8. Wang J, Zhang K, Xu L, Wang EK (2011) Quantifying the waddington landscape and biological paths for development and differentiation. Proc Natl Acad Sci USA 108: 8257-8262.

9. McQuarrie DA (1983) Quantum Chemistry. California: University Science Books.

10. Swain PS, Elowitz MB, Siggia ED (2002) Intrinsic and extrinsic contributions to stochasticity in gene expression. Proc Natl Acad Sci U S A 99: 12795-12800.

11. Kaern M, Elston TC, Blake WJ, Collins JJ (2005) Stochasticity in gene expression: from theories to phenotypes. Nat Rev Genet 6: 451-64.

12. Hunt K, Ross J (1981) Path integral solutions of stochastic equations for nonlinear irreversible processes: The uniqueness of the thermodynamic lagrangian. J Chem Phys 75: 976-984.

13. Wang J, Zhang K, Wang EK (2010) Kinetic paths, time scale, and underlying landscapes: A path integral framework to study global natures of nonequilibrium systems and networks. J Chem Phys 133: 125103:1-13.

14. Faccioli P, Sega M, Pederiva F, Orland H (2006) Dominant pathways in protein folding. Phys Rev Lett 97: 1-4.
